# miR-448 targets IDO1 and regulates CD8^+^ T cell response in human colon cancer

**DOI:** 10.1186/s40425-019-0691-0

**Published:** 2019-08-07

**Authors:** Qiong Lou, Ruixian Liu, Xiangling Yang, Weiqian Li, Lanlan Huang, Lili Wei, Huiliu Tan, Nanlin Xiang, Kawo Chan, Junxiong Chen, Huanliang Liu

**Affiliations:** 1 Guangdong Provincial Key Laboratory of Colorectal and Pelvic Floor Diseases, Guangdong Institute of Gastroenterology, The Sixth Affiliated Hospital, Sun Yat-sen University, Guangzhou, 510655 Guangdong China; 2 Department of Clinical Laboratory, The Sixth Affiliated Hospital, Sun Yat-sen University, Guangzhou, 510655 Guangdong China

**Keywords:** IDO1, miR-448, CD8, Colon cancer, Immunology, Tumor microenvironment

## Abstract

**Background:**

Indoleamine 2,3-dioxygenase 1 (IDO1) is a critical regulator of T cell function, contributing to immune tolerance. Upregulation of IDO1 has been found in many cancer types; however, the regulatory mechanisms and clinical significance of IDO1 in colon cancer are still unclear. Here, we investigated the role of dysregulated microRNA (miRNA) targeting IDO1 in the colon cancer microenvironment.

**Methods:**

We elucidated IDO1 function by performing cell-based assays and establishing transplanted tumor models in BALB/c mice and BALB/c nude mice. We evaluated IDO1 protein expression by immunohistochemistry (IHC) in a tissue microarray (TMA) and analyzed IDO1 mRNA expression with The Cancer Genome Atlas (TCGA). We screened miRNAs targeting IDO1 by using a dual luciferase reporter assay. We tested the function of microRNA-448 (miR-448) by using western blotting (WB) and fluorescence-activated cell sorting (FACS).

**Results:**

We demonstrated that stable IDO1 overexpression enhanced xenograft tumor growth in BALB/c mice but not in BALB/c nude mice. We also revealed the involvement of posttranscriptional regulation of IDO1 in colon cancer by observing IDO1 protein levels and mRNA levels. Furthermore, ectopic expression of miRNA mimics suggested that miR-448 could significantly downregulate IDO1 protein expression. Notably, we proved that miR-448 suppressed the apoptosis of CD8^+^ T cells by suppressing IDO1 enzyme function.

**Conclusion:**

Our findings indicated that IDO1 suppressed the CD8^+^ T cell response in colon cancer. miR-448, as a tumor-suppressive miRNA, enhanced the CD8^+^ T cell response by inhibiting IDO1 expression. The results provide a theoretical basis for the development of new immunotherapy for the treatment of colon cancer.

**Electronic supplementary material:**

The online version of this article (10.1186/s40425-019-0691-0) contains supplementary material, which is available to authorized users.

## Background

Colorectal cancer (CRC) is the third most common cancer worldwide and the second most common cancer-associated mortality [1]. Currently, immunotherapy may provide an ideal approach for patients with microsatellite instability-high (MSI-H) metastatic colon cancer [2–6]. Exploiting tumor cell-intrinsic checkpoint expression is revolutionizing cancer immunotherapy by inducing meaningful clinical responses in many cancer types; some examples of these therapies include targeting PD1 and PDL1 [5, 7]. Other studies have confirmed that the tumor microenvironment has more inhibitory factors, including indoleamine 2,3-dioxygenase 1 (IDO1) [8].

IDO1 is an immunomodulatory enzyme that catalyzes the degradation of tryptophan (Trp) to kynurenine (Kyn). The depletion of Trp and accumulation of Kyn have been reported to induce effector T cell apoptosis/dysfunction and generate immunosuppressive regulatory T cells [9]. Recently, functional inactivation of tumor-reactive T cells has been considered to be a vital mechanism of tumor immune evasion [10]. However, whether IDO1 affects the quantity of tumor-infiltrating T lymphocytes in colon cancer is not clearly defined. G. Brandacher et al. reported that higher IDO1 expression significantly decreased CD3^+^ T lymphocytes in human CRC [11]. However, L. Ferdinande et al. reported that there was no significant correlation between the level of IDO1 expression and the number of CD3^+^ or CD8^+^ T lymphocytes [12]. In addition, CD8^+^ T cells produce cytokines such as interferon-*γ* (IFN-*γ*) [13]. IDO1 is strongly induced by IFN-*γ* in the tumor cells [14]. Therefore, our studies aim to examine the correlation of IDO1 expression and CD8^+^ T lymphocyte infiltration in colon cancer.

MicroRNAs (miRNAs) act as intrinsic mediators in a variety of biological processes, such as cancer development, angiogenesis and the immune response, by downregulating gene expression at the posttranscriptional level [15]. Recent studies have shown that miRNAs are aberrantly expressed in colon cancer and are involved in the regulation of immune escape in colon cancer [16–19]. Additionally, IDO1 is reported to be highly expressed in a wide variety of human cancers [20]. We suggest that there may be important endogenous miRNAs targeting IDO1. These miRNAs may downregulate IDO1 expression at the posttranscriptional level and affect the CD8^+^ T cell response in the colon cancer microenvironment. A previous study found that miR-153 targeted IDO1 in graft-versus-host disease and colon cancer [19, 21], and miR-448 targeted IDO1 in breast cancer [22]. However, there are no reports about miRNA targeting IDO1 in colon cancer and how miRNAs affect the T cell response via IDO1 in the colon cancer microenvironment is less well characterized.

In this study, we investigated the role of IDO1 in the tumor microenvironment by injecting CT26 cells with stable IDO1 overexpression into immune-competent mice. We examined the changes in the angiogenesis, proliferation, and apoptosis of tumor cells as well as natural killer (NK) cell and T lymphocyte responses by IHC in subcutaneous tumor tissues. In addition, we performed the dual luciferase reporter assay and WB assay and found that miR-448 targeted IDO1. We confirmed by FACS that miR448 can affect CD8^+^ T cells by regulating IDO1 in human peripheral blood. The intent was to provide a theoretical basis for the development of new immunotherapy for the treatment of colon cancer.

## Methods

### Human samples

Human colon cancer tissue microarray (TMA) slides, which contain 100 colon cancer tissues and 60 adjacent noncancerous tissues, were purchased from Shanghai Outdo Biotech Inc. (Cat# HColA160Su02, China) and used for immunohistochemical analysis of IDO1 expression. Another TMA (Cat# HColA160Su02) was used for in situ hybridization (ISH) analysis of miR-448 expression. Peripheral blood samples from healthy adult volunteers were collected at The Sixth Affiliated Hospital of Sun Yat-sen University (Guangzhou, China). The blood samples were collected after obtaining the patients’ informed consent according to the local ethical committee.

### Cell culture

The human colon cancer cell lines HCT-116 and HT-29 and the mouse colon cancer cell line CT26 were maintained in RPMI 1640 (Gibco, USA) containing 10% fetal bovine serum (FBS, Gibco) at 37 °C under 5% CO_2_. All cell lines were obtained from American Type Culture Collection (ATCC).

### IDO1 stable overexpression

IDO1 was cloned and inserted into the lentiviral expression vector CMV-T7-MCS-SV40-IRES-Puro (Guangzhou HYY Medical Science Limited Company, China). CT26 cells stably overexpressing IDO1 (pLenti-IDO1) and vector control (pLenti-Vector) were selected for over 2 weeks by adding the minimum concentration of puromycin. The efficacy of stable IDO1 overexpression was detected by quantitative real-time polymerase chain reaction (qRT-PCR) and WB.

### Animal studies

Male BALB/c mice and male BALB/c nude mice (4–5 weeks) were purchased from Model Animal Research Center of Nanjing University. The mice were kept in a specific pathogen-free environment under isothermal conditions with regular photoperiods. All animal studies were approved by the Animal Ethical and Welfare Committee of Sun Yat-sen University (IACUC DD-17-1101). The mice were randomly divided into 2 groups (*n* = 6) and subcutaneously inoculated in the right flank with 4 × 10^5^ CT26 cells with or without stable IDO1 overexpression. Tumor volume was measured with a digital caliper (π/6 × length × width^2^), and body weight was periodically recorded. Mice were sacrificed when tumors reached 2.0 cm in diameter.

### Transwell assay

In the transwell cell migration assay, 24-well inserts from Corning were used. In brief, 1 × 10^5^ CT26 cells in 200 μL of serum-free medium were added into the upper chamber, while 600 μL of medium containing 10% FBS was placed into the lower chamber. After 24 h, cells remaining in the upper surface of the membrane were carefully wiped off, and migratory cells on the lower surface were washed. Before observation, the cells were fixed with methanol and stained with crystal violet for 15 min. Transwell chambers precoated with Matrigel (BD Bioscience) were used to conduct the invasion assay in the same manner as the migration assay.

### Real-time cell analysis

5 × 10^3^ cells/well of CT26 cells with or without stable IDO1 overexpression were seeded into a 96-well E-plate (ACEA Biosciences). The impedance was detected using the xCELLigence real-time cell analysis (RTCA) system according to manufacturer’s recommendations. For each plot, the *Y*-axis represents the normalized cell index, and the *X*-axis represents time in hours. All impedance assays were repeated at least 3 times to ensure reproducibility.

### Wound healing assay

The wound healing assay was performed as described [23]. The two-well culture insert (Ibidi GmbH, Germany) was placed in the middle of a 6-well plate. CT26 cells were adjusted to 7 × 10^5^ cells/mL in complete medium; 70 μL of the suspension were added into two reservoirs of the culture insert. We then removed the culture insert and added 2 mL of complete medium into the well after cells were allowed to adhere and grow overnight. Wound closure was monitored using a light microscope over a period of 18 h and the relative wound areas were analyzed using ImageJ software. Each assay was repeated three times.

### Immunofluorescent staining

Paraffin mouse tumor tissue sections were pre-treated using heat mediated antigen retrieval with sodium citrate buffer (pH 6.0) for 30 min, and blocked with normal goat serum (Cat# AR0009, Boster, China) for 1 h at room temperature. Then, the slides were performed with rat anti-mouse IDO1 (1:100, Cat# 122402, BioLegend), and rabbit anti-mouse CD8 antibody (1:200, Cat# ab203035, Abcam) overnight at 4 °C. After the slides were washed with PBS, they were incubated with Alexa Fluor 633-conjugated anti-rat (1:200, Cat# A-21094, Invitrogen) or Alexa Fluor 594-conjugated anti-rabbit (1:200, Cat# R37117, Life Technologies) secondary antibodies for 2 h in the dark at room temperature. These antibodies were used for labeling the rat anti-IDO1 antibody or the rabbit anti-CD8 antibody, respectively. Slides were mounted using VECTASHIELD antifade mounting medium containing 4,6-diamidino-2-phenylindole (DAPI) (Cat# H-1200, Vector Laboratories) according to manufacturer’s recommendations. Fluorescent staining was visualized under a laser scanning confocal microscope (TCS SP8, Leica, Germany).

### Immunohistochemical staining

The following primary antibodies were used for IHC: rat anti-mouse IDO1 (1:1000, Cat# 122402) from BioLegend; rabbit anti-mouse CD8 (1:200, Cat# ab203035), rabbit anti-mouse CD4 (1:500, Cat# ab183685), rabbit anti-NCR1 (1:200, Cat# ab214468) and rabbit anti-Ki67 (1:400, Cat# ab21700) from Abcam; rabbit anti-CD31 (1:100, Cat# 77699) and rabbit anti-human IDO1 (1:100, Cat# 86630) from Cell Signaling Technology (CST). Slides were incubated with primary antibodies at 4 °C overnight. The remaining steps were performed according to horseradish peroxidase-conjugated secondary antibody instructions (Cat# PV-9004 or PV-9001, Zhongshan Jinqiao Biotechnology Co., Ltd. China). The slides were developed with diaminobenzidine (DAKO) and counterstained with hematoxylin (Sigma). TUNEL staining in tumor tissues was detected using a TUNEL-POD kit (Cat# KGA7052, KeyGen Biotech, China) according to the manufacturer’s instructions. Positively stained cells in 5 randomly selected fields were counted, and the mean number of positive cells per field was calculated. Staining of IDO1, CD8 and CD4 (T cell marker panels), NCR1 (a pan NK marker), CD31 (a tumor microvessel marker), Ki67 (a proliferation marker) and TUNEL (an apoptosis marker) was quantitatively analyzed by Image-Pro Plus 6.0.

### Oligonucleotide transfection

miRNA mimics and a negative control (NC) were synthesized by RiboBio (Guangzhou, China). Transfection of HT-29 and HCT-116 cells was performed using Lipofectamine® RNAiMAX (Invitrogen) as described [24]. After these treatments, the cells were harvested for WB assays.

### Quantitative RT-PCR

Total RNA was extracted from cells using TRIzol (Invitrogen) and transcribed to cDNA using a cDNA reverse transcription kit (Takara, Dalian, China). The primer sequences were shown in Additional file 1: Table S1. qRT-PCR was performed using a LightCycler® amplification and detection system (Roche, Switzerland). Differences in the gene expression levels between two groups were evaluated using an independent t-test, and *P* < 0.05 was considered significant.

### Luciferase reporter assay

The full-length IDO1 3′ UTR sequence was cloned into pmirGLO vector downstream of the firefly luciferase gene, which was constructed by Sangon Biotech (Shanghai, China). The Renilla luciferase gene was expressed as a reference reporter in the pmirGLO vector. IDO1 was transfected into 1 × 10^5^ HCT-116 or HT-29 cells along with 40 miRNA mimics or a miRNA NC (RiboBio, China) in 24-well plates. At 24 h posttransfection, cell lysates were assayed for luciferase activity using Dual-Luciferase Assay system (Promega) according to manufacturer’s instructions.

### Western blotting assay

Protein extracted from cells and tissue samples was analyzed by WB. The following primary antibodies were used: rabbit anti-human IDO1 (1:1000, Cat# 86630, CST), rat anti-mouse IDO1 (1:1000, Cat# 122402, BioLegend), rabbit GAPDH (1:10000, Cat# 10494–1-AP, Proteintech). Anti-mouse and anti-rabbit secondary antibodies were purchased from Santa Cruz Biotechnology (1:5000), and anti-rat secondary antibody was purchased from BioLegend (1:5000, Cat# 405405). Densitometric analysis of protein blots were analyzed using ImageJ (NIH, Bethesda, MD). The value of IDO1 intensity was then normalized to the value of corresponding GAPDH intensity and was displayed as a percentage of the value of pLenti-Vector samples obtained in the same way (considered as 100%).

### In situ hybridization of miR-448

ISH was performed using a hsa-miR-448 probe from Boster (3′-DIG labeled). Detection of probe was carried out using an ISH detection kit (Cat# MK10591, Boster, China) according to the manufacturer’s protocol. ISH staining of the image was analyzed using Image-Pro-Plus 6.0.

### Determination of IDO1, tryptophan and kynurenine

The concentration of IDO1 from culture medium was detected by ELISA (Cat# JL20020, JiangLai, China) according to the manufacturer’s protocol. The concentration of Trp (Cat# BWB51529, National Institutes for Food and Drug Control) and Kyn (Cat# K8625, Sigma) from culture medium were measured by using high performance liquid chromatography (HPLC). The samples were separated on a Welch Ultimate AQ-C18 column (250 mm × 4.6 mm, 5 μm) with a mobile phase of acetonitrile and 15 mM potassium phosphate buffer (8:92, v/v) at a flow rate of 1.0 mL/min with a column temperature 35 °C. Potassium phosphate buffer was adjusted to pH 3.6 with acetic acid. The UV detection wavelengths for Trp and Kyn are 280 nm and 360 nm, respectively. Thirty microliters of HT-29 and HCT-116 cell lysate was used to assess IDO1 function according to the manufacturer’s protocol (IDO1 Activity Assay Kit, Cat# K972, Biovision).

### Isolating primary human leukocytes

Human leukocytes were isolated from peripheral blood and collected into tubes containing ethylenediaminetetraacetic acid for immediate analysis. Blood samples were centrifuged at 500×g for 10 min at room temperature, and the supernatant was discarded. Then, 10 times the volume of ACK lysis buffer (Cat# CS0001, Leagene) was added, and the samples were gently vortexed for 5 min. After centrifugation (500×g, 10 min), the supernatant was discarded. We repeated the above steps until all red blood cells were completely removed. Lastly, we obtained leukocyte and adjusted to 4 × 10^6^ cells/mL in RPMI 1640 containing 10% FBS.

### Primary human leukocyte cell culture

miR-448 mimic or NC was transfected into HT-29 and HCT-116 cells in the presence or absence of 50 ng/mL or 250 ng/mL IFN-*γ* (GenScript, China), respectively. We collected the cell culture supernatant after treatment with IFN-*γ* for 24 h as the conditional medium (CM). One hundred microliters of leukocytes were seeded into a 96-well plate, after which 100 μL of different CM was added into the 96-well plate and cultured for 48 h.

### Flow cytometry analysis

Leukocytes were collected after 48 h of culture in different CMs, suspended in PBS, and stained with FITC anti-human CD8 (Cat# 344704, BioLegend) for 15 min at 4 °C in the dark. Cells were centrifuged and suspended in binding buffer, and subjected to Annexin V-APC/7-AAD double staining according to the manufacture’s instructions (Cat# AP105, Multi Sciences). Mouse tumor tissues were dissociated into single-cell suspensions using the tumor dissociation kit of mouse (Cat# 130–096-730, Miltenyi). Single-cell suspensions were stimulated with BD leukocyte activation cocktail (Cat# 550583) for 5 h. Tumor cells were stained with Zombie Yellow™ fixable viability kit for 0.5 h. Cells were then stained with CD45, CD4, CD8, PD-1, Tim3 and Lag3 for 0.5 h. After fixation and permeabilization (Cat# 00–5523-00, eBioscience™), cells were fluorescently stained with Foxp3, T-bet, Perforin, Granzyme B and IFN-*γ*. All antibodies for flow cytometry analysis of mouse tumor samples were shown in Additional file 1: Table S2. All samples were run through a Cytek Aurora flow cytometer and analyzed with FlowJo software.

### Statistical analysis

Two-tailed Student’s t-test and *χ*^2^ tests were used to compare two independent groups. One-way analysis of variance (ANOVA) was used when more than two groups were compared. Survival functions were estimated by Kaplan–Meier methods and compared using the log-rank test. All analyses were performed using SPSS 16.0 software or GraphPad Prism v.8.0 software. *P* < 0.05 was considered significant. Unless noted, samples were independent biological replicates.

## Results

### Overexpression of IDO1 has no effect on migration, invasion, colony formation and proliferation of CT26 cells

**Fig. 1:**
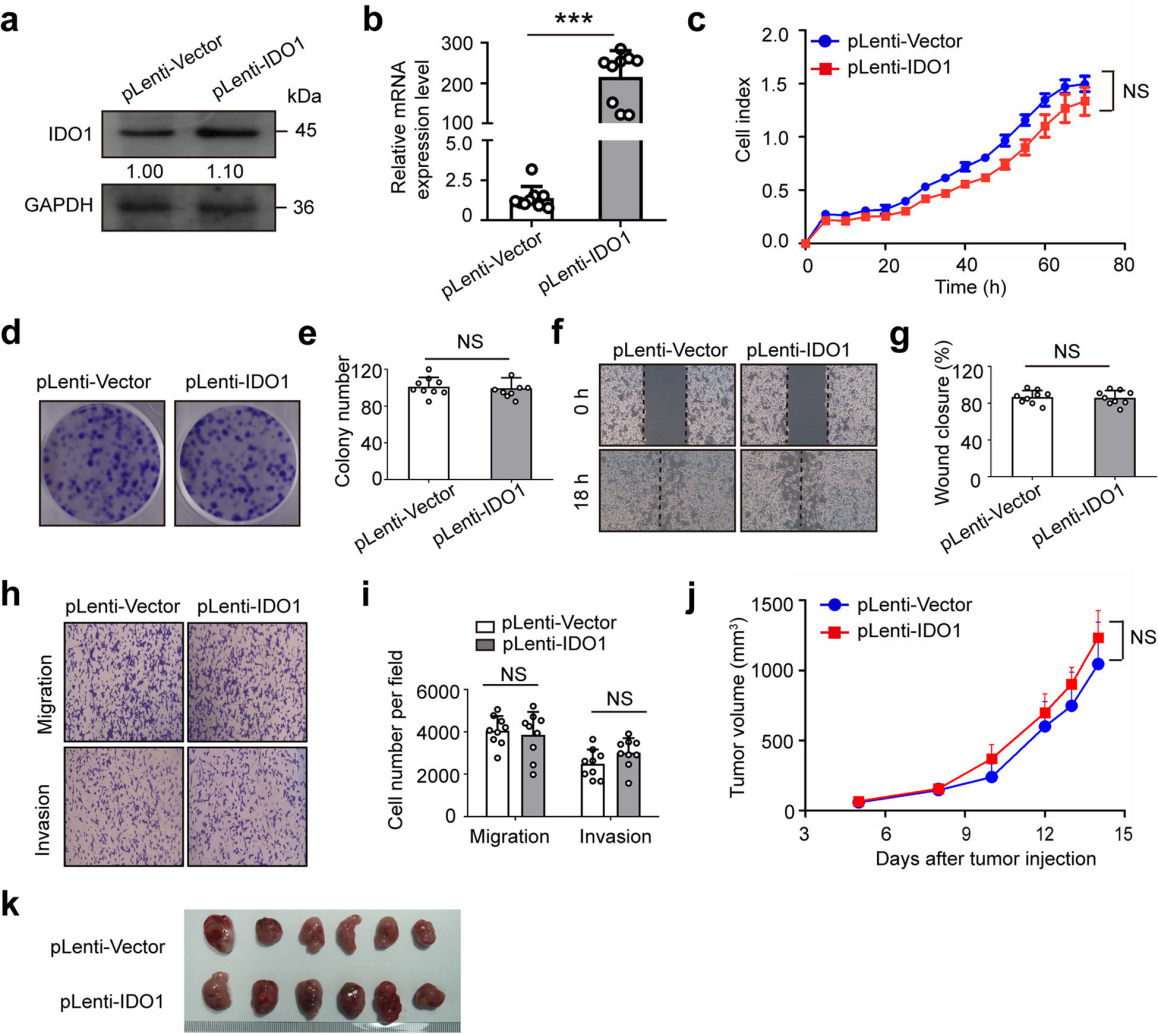
IDO1 overexpression has no effect on the migration, invasion and proliferation of CT26 cells. **a** Representative western blots and **b** qRT-PCR results of IDO1 in CT26 cells from the stable IDO1 overexpression (pLenti-IDO1) group and the vector control (pLenti-Vector) group. GAPDH was served as the internal control. **c** Representative pictures of real-time proliferation assay in CT26 cells from the pLenti-IDO1 group and the pLenti-Vector group. **d** Representative pictures and **e** quantitative data of colony formation assay in CT26 cells from the pLenti-IDO1 group and the pLenti-Vector group. **f** Representative pictures and **g** quantitative data of wound healing assay in CT26 cells from the pLenti-IDO1 group and the pLenti-Vector group. **h** Representative pictures and **i** quantitative data of transwell assay in CT26 cells from the pLenti-IDO1 group and the pLenti-Vector group. **a**, **c**, **d**, **f**, **h** Images are representative of three biological replicates. **j** Average growth curves of subcutaneous xenograft tumors in BALB/c nude mice after inoculation of CT26 cells with pLenti-IDO1 or with pLenti-Vector. **k** Representative pictures of tumors in BALB/c nude mice from the pLenti-IDO1 group and the pLenti-Vector group. **b**, **c**, **e**, **g**, **i**, **j** Mean ± SEM. **b**, **e**, **g**, **i** *n* = 9 measurements from three biological replicates performed in triplicate; **c** *n* = 3; **j**, **k** *n* = 6. **b**, **c**, **e**, **g**, **i** Two-tailed Student’s t-test and **j** one-way ANOVA were performed for statistical analysis; ***P < 0.001, NS: not significant

To determine whether IDO1 affects colon cancer cell phenotypes, we established stable IDO1 overexpression of CT26 cells and performed a series of in vitro cell-based assays. The efficacy of overexpression was validated by WB and qRT-PCR assays (Fig. 1a and b). Next, RTCA results showed that stable IDO1 overexpression did not enhance cell growth in CT26 cells (Fig. 1c). Similarly, the colony formation assay indicated that CT26 cells stably overexpressing IDO1 had neither more nor larger colonies following a 10-day incubation than vector control cells (Fig. 1d and e). In addition, the migratory and invasive activities of CT26 cells were not obviously changed by stable IDO1 overexpression (Fig. 1f-i). These results implied that IDO1 had no effect on colon cancer cell phenotypes in vitro. Then, we subcutaneously inoculated CT26 cells with or without stable IDO1 overexpression into immune-deficient BALB/c nude mice. The results showed that IDO1 had no effect on the growth of CT26 tumors in immune-deficient nude mice in vivo (Fig. 1j and k).

### Stable IDO1 overexpression enhances xenograft tumor growth in BALB/c mice

**Fig. 2:**
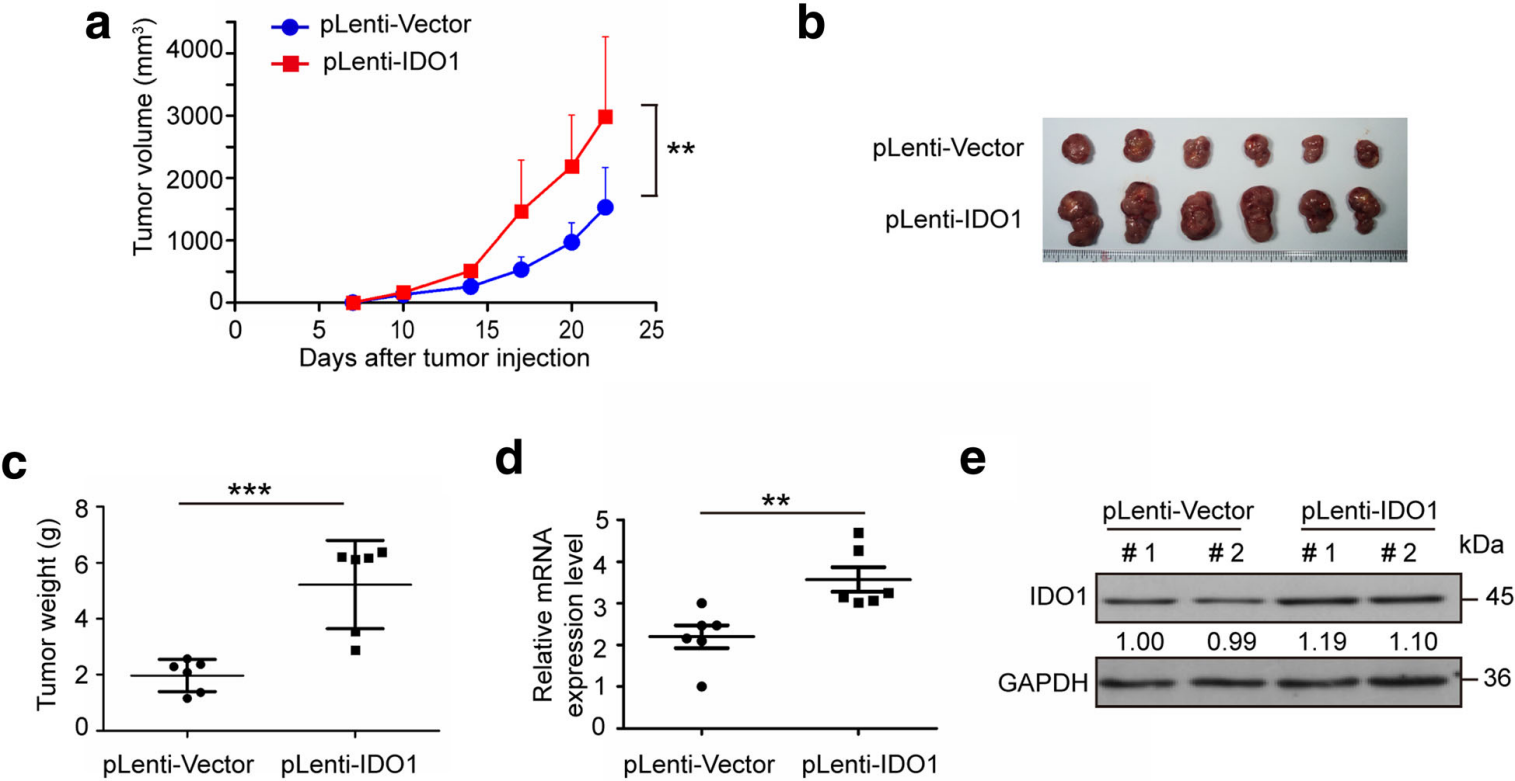
Stable IDO1 overexpression enhances xenograft tumor growth in BALB/c mice. **a** Average growth curves of subcutaneous xenograft tumors in BALB/c mice after inoculation of CT26 cells with stable IDO1 overexpression (pLenti-IDO1) or with vector control (pLenti-Vector). **b** Representative pictures of tumors in BALB/c mice from the pLenti-IDO1 group and the pLenti-Vector group. **c** Tumor weight in mice from the pLenti-IDO1 group and the pLenti-Vector group**. d** qRT-PCR results and **e** representative western blots of IDO1 in subcutaneous tumor tissues from the pLenti-IDO1 group and the pLenti-Vector group. # 1 and # 2 represents tumor tissues from different mice. Images are representative of three biological replicates. **a** Mean ± SEM; **c**, **d** mean ± SD. **a-d** *n* = 6. **a** One-way ANOVA and **c**, **d** two-tailed Student’s t-test were performed for statistical analysis; **P < 0.01, ***P < 0.001

Having identified the anti-tumor effects of CT26 cells with IDO1 overexpression in immune-deficient nude mice, we next examined the role of IDO1 in the tumor microenvironment in immune-competent mouse model. We subcutaneously inoculated CT26 cells with or without stable IDO1 overexpression into immune-competent BALB/c mice. The tumors from IDO1 overexpression group showed increased volume and weight compared with tumors from the vector control group (Fig. 2a-c). In addition, the efficacy of mRNA and protein overexpression in mice tumors was validated by qRT-PCR and WB assays (Fig. 2d and e). There was no significant difference in mice body weight between two groups in BALB/c mice (Additional file 1: Figure S1a). These results indicated that IDO1 may contribute to xenograft tumor growth in BALB/c mice.

### IDO1 suppresses the CD8^+^ T cell response in subcutaneous tumor tissue

**Fig. 3:**
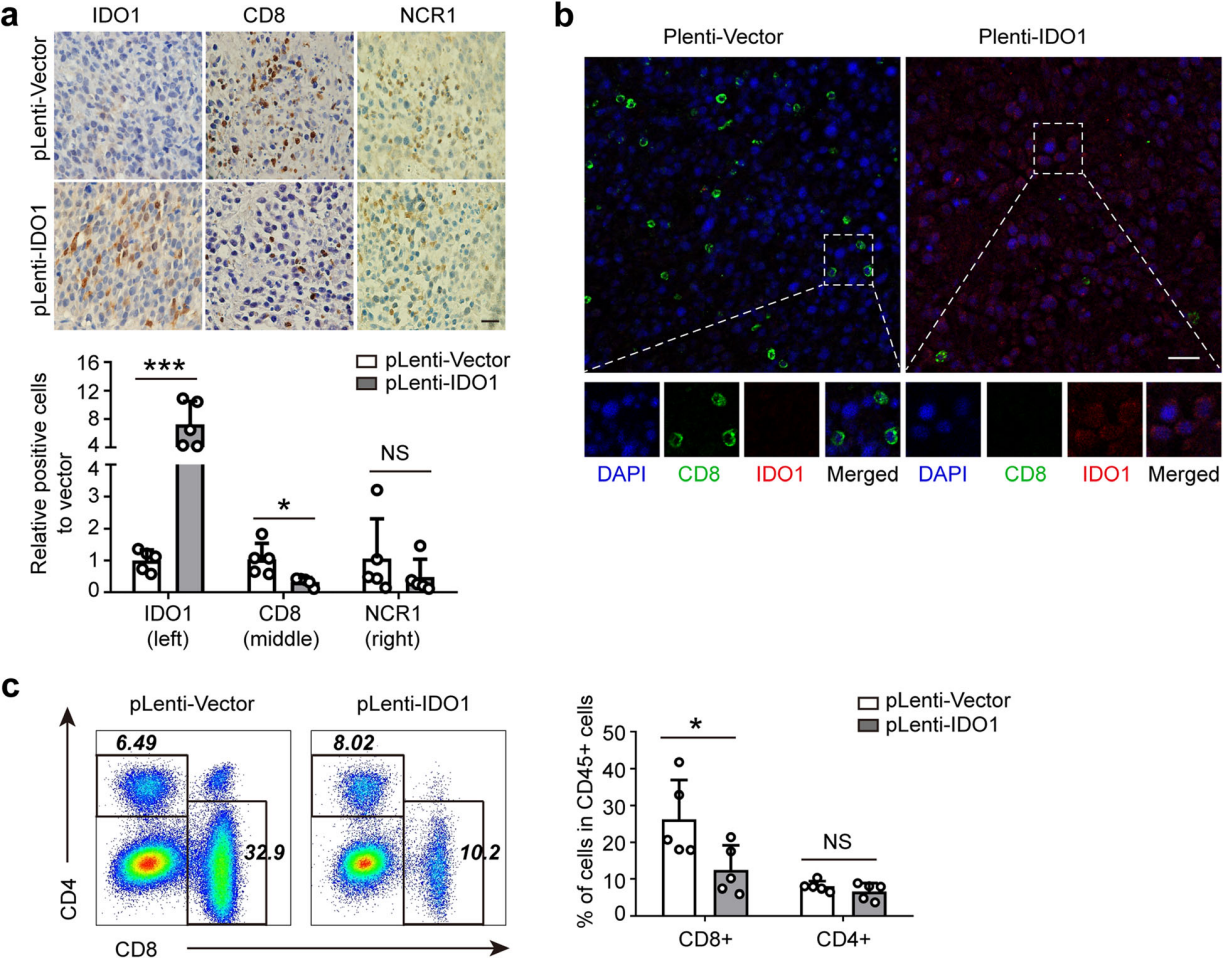
IDO1 suppresses the CD8^+^ T cell response in subcutaneous tumor tissue. **a** Immunohistochemical staining for IDO1, CD8 and NCR1 in sections of mouse tumor tissues from the pLenti-IDO1 group and the pLenti-Vector group. Top, representative pictures; bottom, quantitative data. Data represent the relative percentage of IDO1^+^, CD8^+^ and NCR1^+^ cells in the pLenti-IDO1 group to that in the pLenti-Vector group. Scale bars: 20 μm. **b** Representative pictures of immunofluorescent staining for IDO1 expression tumor cells and infiltrating CD8^+^ lymphocytes in sections of mouse tumor tissues from the pLenti-IDO1 group and the pLenti-Vector group. Blue, DAPI staining; red, IDO1 staining; green, CD8 staining. Scale bars: 20 μm. **c** FACS analysis of CD8^+^ T cells and CD4^+^ T cells in CD45^+^ cells of mice tumor tissues in the pLenti-IDO1 group and pLenti-Vector group. Left, representative pictures; right, quantitative data. **a** Mean ± SEM; **c** mean ± SD. **a-c** *n* = 5. **a**, **c** Two-tailed Student’s t-test was performed for statistical analysis; *P < 0.05, ***P < 0.001, NS: not significant

Given that IDO1 overexpression promoted tumor growth in tumor-bearing BALB/c mice in vivo, we used IHC techniques to further explore the potential mechanism by which stable IDO1 overexpression drives subcutaneous tumor growth in vivo. The following hypothetical mechanisms were considered to explain the above phenomenon: changes in tumor microvessel formation (CD31) [25], tumor proliferation (Ki67) [26], tumor apoptosis (TUNEL) [27], the NK cell response (NCR1) [28] or the T lymphocyte response (CD8 and CD4) [29]. As a result, the numbers of IDO1^+^ cells were significantly higher in the stable IDO1 overexpression group than in the vector control group (Fig. 3a, left panel). Meanwhile, the number of CD8^+^ T cells in tumor tissues of IDO1 overexpression group was significantly less than that in the vector control group (Fig. 3a, middle panel). However, the expression of NCR1 was increased in IDO1 overexpression group, but the difference did not reach statistical significance (Fig. 3a, right panel). The expression of IDO1 was located in the cytoplasm and was mainly confined to tumor cells, while the expression of CD8 was located on the membrane of lymphocytes (Fig. 3b). Moreover, there was no significant difference in the number of CD4^+^ T cells, CD31^+^ cells, Ki67^+^ cells and TUNEL^+^ cells between the stable IDO1 overexpression group and the vector control group (Additional file 1: Figure S1b and c).

Then, we used FACS to evaluate the proportion of CD8^+^ T cells and CD4^+^ T cells in CD45^+^ cells and detect the phenotype of CD8^+^ T cells and CD4^+^ T cells. The results showed that the proportion of CD8^+^ T cells in CD45^+^ cells was significantly lower in the stable IDO1 overexpression group than in the vector control group (Fig. 3c). However, there was no significant difference in the proportion of CD4^+^ T cells, T-bet^+^ cells, Foxp3^+^ cells, IFN-*γ*^+^ cells, granzyme B^+^ cells, perforin^+^ cells, PD-1^+^ cells, Lag3^+^ cells and Tim3^+^ cells between the two groups (Fig. 3c and Additional file 1: Figure S2). Thus, these results indicated that IDO1 contributes to immune escape of CT26 cells in BALB/c mice by mainly suppressing the CD8^+^ T cell response.

### The protein and mRNA expression pattern of IDO1 are different in human colon cancer

**Fig. 4:**
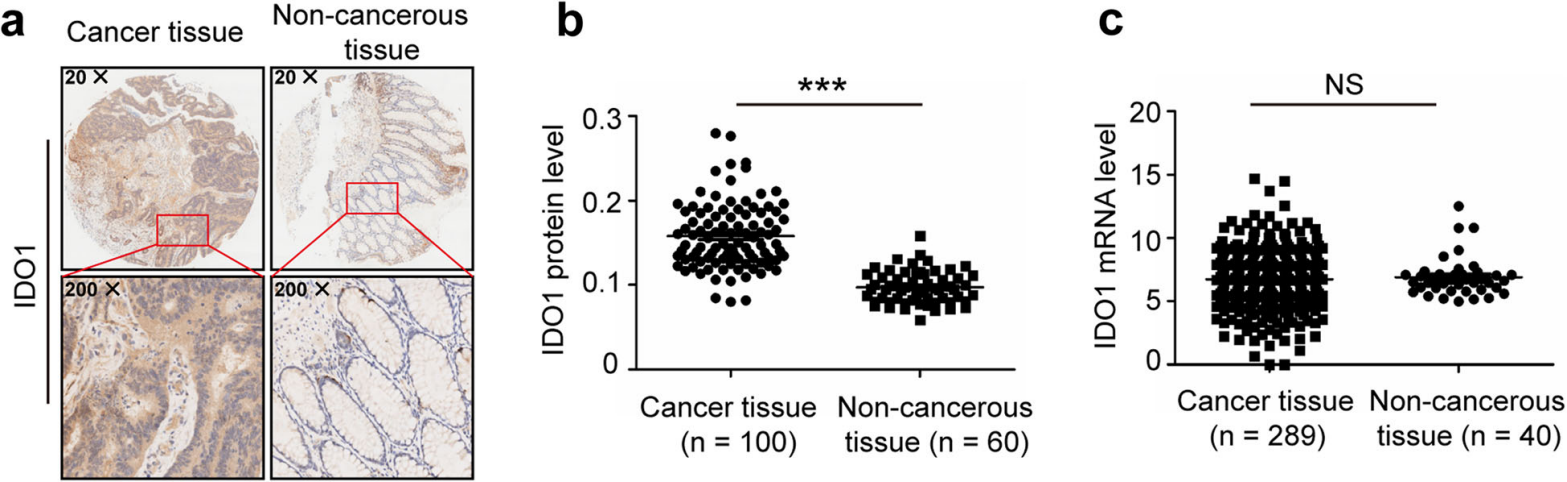
The protein and mRNA expression patterns of IDO1 are different in human colon cancer. **a** Representative IHC staining of IDO1 in human colon cancer tissues and adjacent noncancerous tissues. **b** IDO1 protein expression of 100 colon cancer tissues and 60 adjacent noncancerous tissues by IHC in a tissue microarray. The data were quantified by measuring the mean density of all the DAB stained areas of each micrograph using Image-Pro Plus 6.0 software, and the *Y-*axis for “IDO1 protein level” represents the mean density of DAB staining (integral optical density (IOD) / area of interest (AOI)). **c** IDO1 mRNA expression between 289 colon cancer tissues and 40 adjacent noncancerous tissues. The data were downloaded from TCGA. **b**, **c** Two-tailed Student’s t-test was performed for statistical analysis; ***P < 0.001, NS: not significant

After we demonstrated the importance of IDO1-mediated tumor immunity and the association between IDO1 and CD8 in mice (Figs. 2 and 3), we next analyzed IDO1 expression and function in human colon cancer. We tested IDO1 expression in human colon cancer by IHC in a tissue microarray. The expression of IDO1 in colon cancer tissues was higher than that in adjacent noncancerous tissues by IHC (Fig. 4a and Additional file 1: Figure S3). Staining was predominantly observed in the tumor cells with a cytoplasmic/perinuclear pattern. The protein expression level of IDO1 was higher in colon cancer tissues than that in adjacent noncancerous tissues in patients with colon cancer (Fig. 4b), which is consistent with previously reports [30, 31]. However, there was no significant difference in IDO1 mRNA expression (downloaded from TCGA) between colon cancer tissues and adjacent noncancerous tissues (Fig. 4c). The major clinicopathological characteristics of patients with colon cancer are presented (Additional file 1: Table S3 and S4). Therefore, we suggest that there are important regulators during IDO1 mRNA translation. Because miRNA can control gene expression at the posttranscriptional level by targeting the 3′ UTR, we further studied the regulation of IDO1 gene expression mediated by miRNAs.

### miR-448 downregulates the protein expression of IDO1

To identify putative miRNAs targeting IDO1, various miRNA target prediction programs were used. However, each prediction program provides potential but nonidentical predictions, so it is not clear which program is best. We opted to use TargetScan, miRNA.org and miRDB to screen miRNAs targeting IDO1 and selected 23 candidates. In addition, 17 other miRNAs were donated from our laboratory; thus, 40 miRNAs were chosen for experimental validation.

**Fig. 5:**
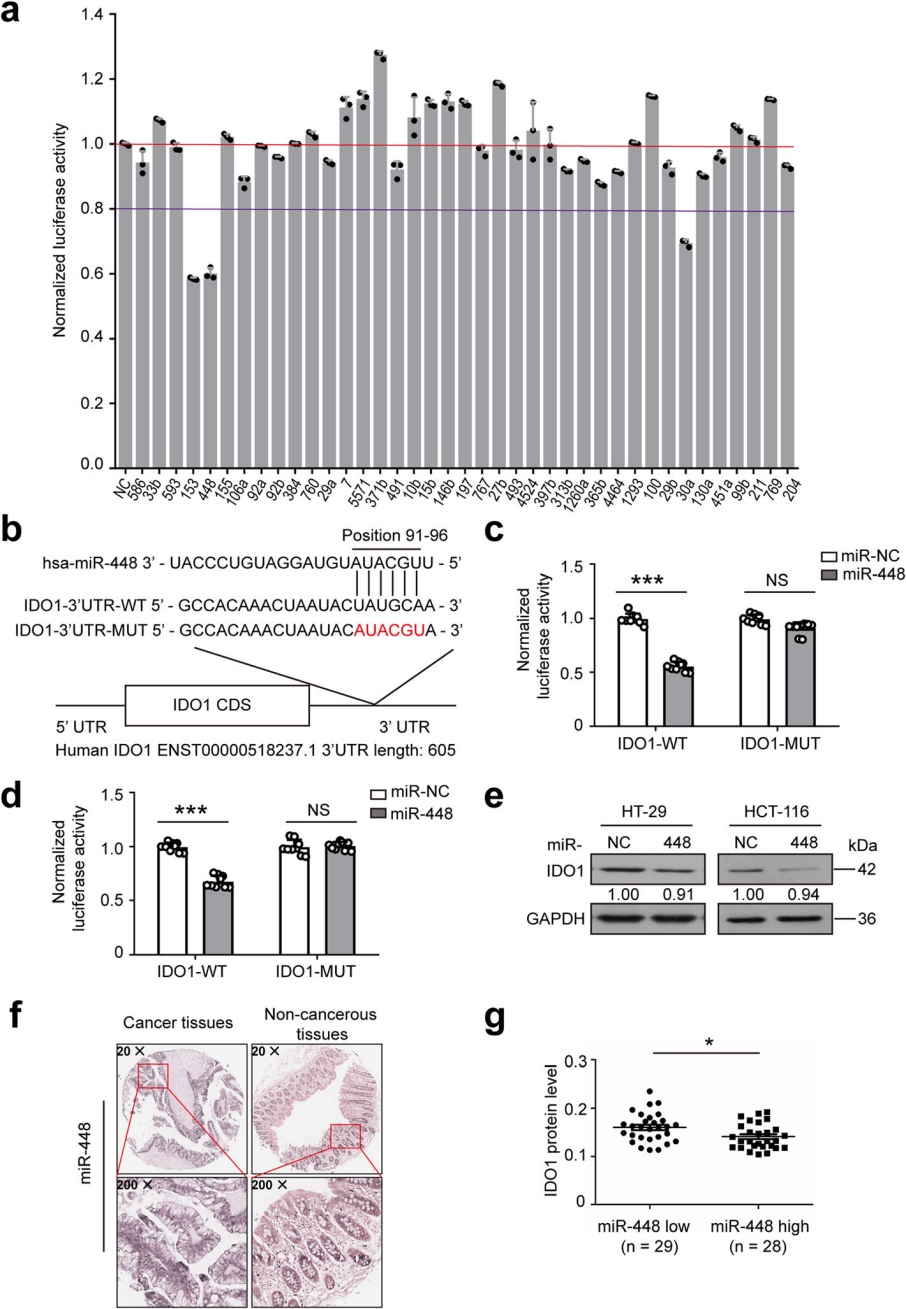
miR-448 downregulates IDO1 expression. **a** Luciferase reporter containing wild-type (WT) IDO1 3′ UTR was cotransfected with 40 miRNA mimics or a negative control (NC) into HCT-116 cells. Relative firefly luciferase expression was normalized to Renilla luciferase. NC: miRNA mimic negative control, 586: miRNA-586 mimic, etc. **b** The binding site of miR-448 in the IDO1 mRNA. The relative luciferase activity in **c** HT-29 cells and **d** HCT-116 cells cotransfected with miR-448 mimics and IDO1-WT, or cotransfected with miR-448 mimics and IDO1-MUT. **e** Representative western blots of IDO1 in HT-29 and HCT-116 cells transfected with miR-NC or miR-448 followed by IFN-*γ* treatment. GAPDH was served as the internal control. Images are representative of three biological replicates. **f** Representative ISH staining of miR-448 in human colon cancer tissues and adjacent noncancerous tissues. **g** IDO1 protein expression in miR-448^low^ group (*n* = 29) and miR-448^high^ group (*n* = 28). miR-448^low^ group and miR-448^high^ group were divided according to the ratio of miR-448 expression in colon cancer tissues to that in adjacent noncancerous tissues (cut-off = 1.33). **a** Mean ± SD; **c**, **d**, **g** mean ± SEM. **c**, **d** *n* = 9 measurements from three biological replicates performed in triplicate. **a**, **c**, **d**, **g** Two-tailed Student’s t-test was performed for statistical analysis; *P < 0.05, ***P < 0.001, NS: not significant. miR-NC: miRNA mimic negative control; miR-448: miRNA-448 mimic

To test whether the 40 selected potential miRNAs target IDO1, the full-length IDO1 3′ UTR sequence was cloned into the pmirGLO vector downstream of the firefly luciferase gene. Forty miRNAs and pmirGLO-IDO1–3′ UTR were introduced into HCT-116 or HT-29 cells. A dual luciferase reporter assay showed that miR-448, miR-30a-5p and miR-153 significantly downregulated luciferase expression in HCT-116 and HT-29 cell lines (Fig. 5a and Additional file 1: Figure S4a). Because miR-153 was reported previously [19], we further studied the other two miRNAs. The base pairing between IDO1 3′ UTR and either miR-448 or miR-30a-5p (Fig. 5b and Additional file 1: Figure S4b) was predicted by TargetScan. When the miR-448 or miR-30a-5p binding site in the IDO1 3′ UTR was mutated, the reporter downregulation by miR-448 or miR-30a-5p was abolished (Fig. 5c and d, and Additional file 1: Figure S4c).

The baseline IDO1 expression is low in vitro. However, the expression of IDO1 is significantly higher in cancer cells treated with IFN-*γ* than in untreated cells [13]. We used various concentrations (0 to 1000 ng/mL) of IFN-*γ* to treat HT-29 and HCT-116 cells for 6 to 24 h. These results were shown in Additional file 1: Figure S5a and b. IDO1 protein expression peaked and plateaued at 24 h treatment with 250 ng/mL IFN-*γ* in HT-29 cells and 50 ng/mL in HCT-116 cells. The expression of IDO1 protein was determined by WB (Additional file 1: Figure S5a and b). IFN-*γ* treatment significantly elevated the IDO1 protein level in HT-29 and HCT-116 cells (Additional file 1: Figure S5c).

To investigate whether miR-448 and miR-30a-5p can downregulate IDO1 protein expression, we transfected miR-448 mimics or a miRNA NC into the HT-29 and HCT-116 cells before being treated with 250 ng/mL or 50 ng/mL IFN-*γ*, respectively, for 24 h. Compared with NC, miR-448 substantially decreased IFN-*γ*-induced IDO1 expression in the two cell lines as determined by WB (Fig. 5e and Additional file 1: Figure S5d), while miR-30a-5p only weakly decreased IDO1 protein expression in HCT-116 and HT-29 cells (Additional file 1: Figure S5e). Therefore, these data suggested that IDO1 was a credible target of miR-448.

To further verify the biological roles of miR-448 in human colon cancer, we performed ISH to evaluate the miR-448 levels in 60 paired colon cancer tissues and adjacent noncancerous tissues from a tissue microarray slides. The positive staining of miR-448 was expressed as blue-violet (Fig. 5f and Additional file 1: Figure S6). Results showed that the protein expression of IDO1 in miR-448^low^ colon cancer samples were higher than that in miR-448^high^ colon cancer samples (Fig. 5g). These results indicated miR-448 suppressed the protein expression of IDO1 in human colon cancer.

### miR-448 suppresses IDO1 function by suppressing IDO1 protein expression

**Fig. 6:**
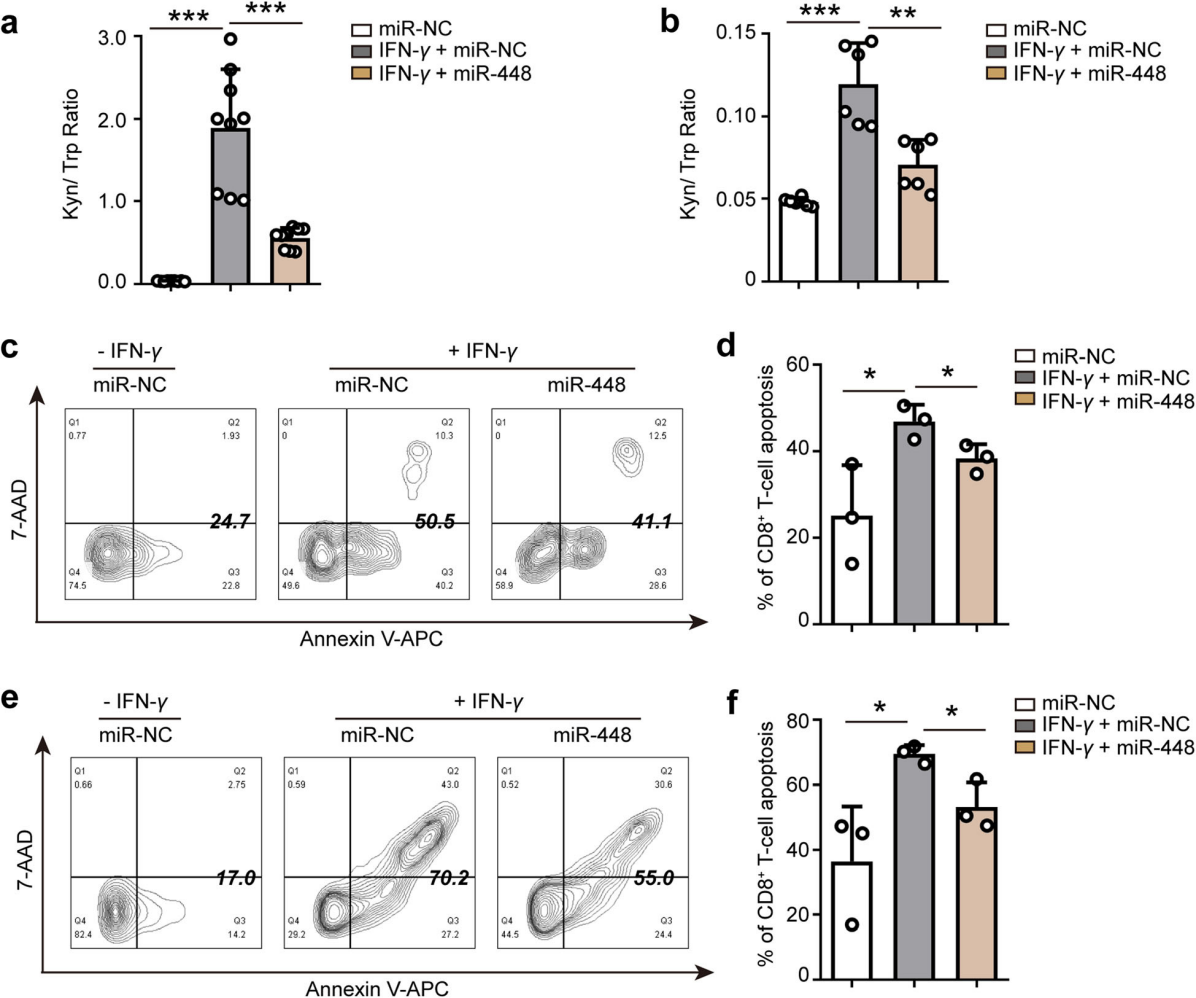
miR-448 enhances the survival of CD8^+^ T cells by suppressing IDO1 function. The Kyn/Trp ratio was analyzed in the culture medium from **a** HT-29 and **b** HCT-116 transfected with miR-NC, or transfected with miR-NC followed by IFN-*γ* treatment, or transfected with miR-448 followed by IFN-*γ* treatment. The concentration of Kyn and Trp were determined by HPLC and the Kyn/Trp ratio was calculated. **c**, **e** Representative contour plots and **d**, **f** apoptosis quantification of FACS analysis for the percentage of apoptotic CD8^+^ T cells in miR-NC, miR-NC + IFN-*γ,* miR-448 + IFN-*γ* conditional medium systems from **c**, **d** HT-29 and **e**, **f** HCT-116 supernatant. **a**, **b**, **d**, **f** Mean ± SEM. **a** *n* = 9 measurements from three biological replicates performed in triplicate; **b** *n* = 6 measurements from two biological replicates performed in triplicate; **c-f** *n* = 3 biologically independent experiments. **a**, **b**, **d**, **f** Two-tailed Student’s t-test was performed for statistical analysis; *P < 0.05, **P < 0.01, ***P < 0.001. miR-NC: miRNA mimic negative control; miR-448: miRNA-448 mimic

IDO1 is mainly expressed in the intracellular of tumor cell, we tried to measure the concentration of IDO1 in the culture supernatant by ELISA (Additional file 1: Table S5). The results showed that the concentration of IDO1 in the culture supernatant samples is low and less than 0.1 IU/mL (Limit of quantification). So it is difficult to directly detect the amount of IDO1 in the culture supernatant of tumor cells. However, IDO1 is an enzyme that catalyzes the degradation of Trp to Kyn. Thus, the Kyn/Trp ratio reflects IDO1 enzyme function. Several methods for detection and quantification of Trp and Kyn have been described using HPLC [32, 33]. Here, we measured the concentration of Trp and Kyn from HT-29 cells or HCT-116 cells culture medium by dual wavelength HPLC (Additional file 1: Figure S7a and b) and analyzed Kyn/Trp ratio in above samples (Fig. 6a and b). The results showed that IFN-*γ* promoted accumulation of Kyn and degradation of Trp, while miR-448 suppressed accumulation of Kyn and degradation of Trp in culture medium from HT-29 cells and HCT-116 cells. IFN-*γ* significantly increased the Kyn/Trp ratio, while miR-448 significantly hampered the Kyn/Trp ratio in culture medium from HT-29 cells and HCT-116 cells. These results indicated that IFN-*γ* promoted IDO1 function, but miR-448 suppressed IDO1 function. Moreover, we assessed IDO1 function in HT-29 and HCT-116 cells lysate by measuring the concentration of Kyn using IDO1 activity assay kit (Additional file 1: Figure S7c and d). The results showed that miR-448 suppressed IDO1 function.

### miR-448 enhances the survival of CD8^+^ T cells by suppressing IDO1 expression

Because miR-448 downregulated IDO1 protein expression and suppressed IDO1 function by targeting IDO1 (Figs. 5e, 6a and b, and Additional file 1: Figure S7), and the IHC results strongly suggested that IDO1 modulated tumor CD8^+^ T cell immunity, it is rational to hypothesize that miR-448 can affect the CD8^+^ T cell response by suppressing IDO1 expression. To our knowledge, CD8^+^ T cells circulate in the blood and exert cytotoxic functions [29]. To verify this hypothesis, we cultured lymphocytes from human peripheral blood samples under different conditions (Additional file 1: Figure S8) for 48 h and analyzed apoptotic CD8^+^ T cells by costaining sample with Annexin V-APC and 7-AAD. We gated lymphocytes, single cells and CD8^+^ T cells and then analyzed the proportion of apoptotic CD8^+^ T cells. The flow cytometry results showed that the group treated with miRNA NC and IFN-*γ* had a significantly higher percentage of apoptotic CD8^+^ T cells than the NC group not treated with IFN-*γ* (Fig. 6c-f). In addition, the miR-448 group showed a significantly reduced percentage of apoptotic CD8^+^ T cells compared to the NC group, indicating that miR-448 enhanced survival of CD8^+^ T cells (Fig. 6c-f). These results showed that miR-448 suppressed the apoptosis of CD8^+^ T cells by blocking IDO1 expression.

## Discussion

In humans, IDO1 is reported to be highly expressed in a wide range of cancers [20]. IDO1 contributes to tumor progression in vivo by suppressing tumor-infiltrating T lymphocytes and NK cells [28, 34] and activating regulatory T cells [35]. The expression of IDO1 in ovarian cancer, brain cancer, melanoma, and acute myeloid leukemia has been found to be a significant predictor of poor prognosis [36–39]. However, the clinical significance of IDO1 expression in colon cancer still seems to be controversial. Some studies have shown that high expression of IDO1 is an adverse prognostic factor in a specific subset of CRC patients [12, 40]. Other studies showed that high IDO1 expression was not significantly correlated with the overall survival of patients [11, 41–43].

Here, our study showed that a high degree of IDO1 expression in colon cancer correlated with a significant reduction in intratumoral CD8^+^ T cells in a mouse model. Human colon cancer constitutively expressed IDO1 and expressed higher IDO1 levels than did corresponding normal tissues by IHC, as previously reported [30, 31]. Furthermore, high levels of IDO1 mRNA and protein expression were not significantly associated with overall survival in colon cancer patients (Additional file 1: Figure S9a and b). Interestingly, high IDO1 protein expression was positively correlated with overall survival in patients with early-stage colon cancer but not in patients with late-stage colon cancer (Additional file 1: Figure S9c and d).

**Fig. 7:**
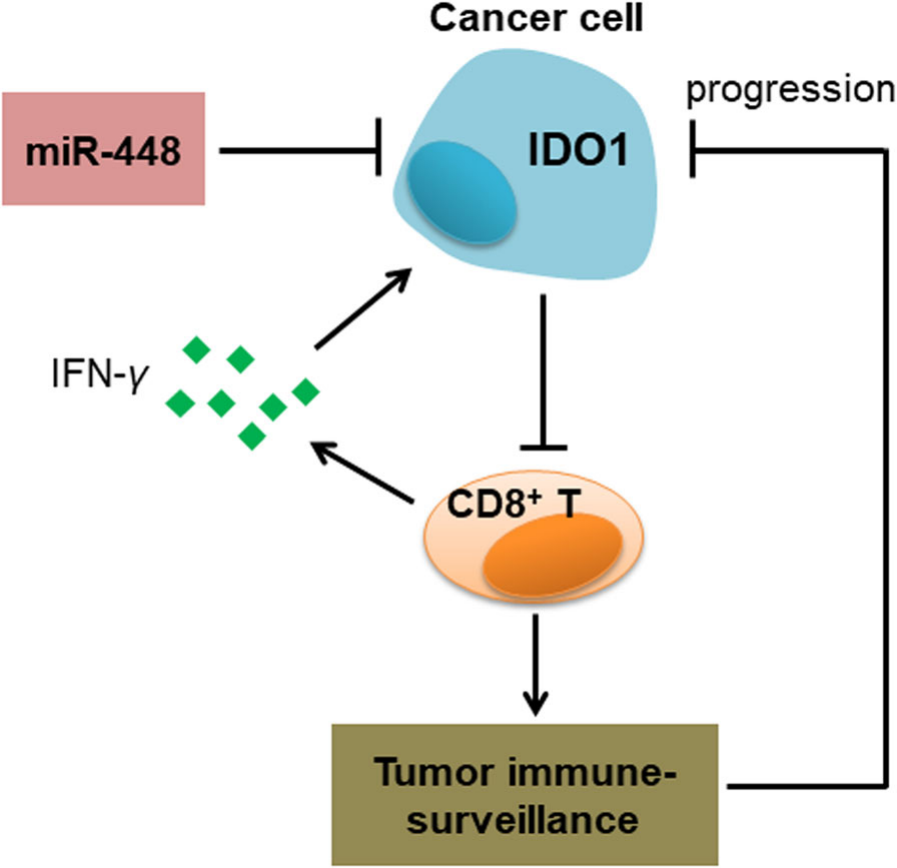
A possible negative feedback loop to regulate CD8^+^ T cell activation. Upregulation of IDO1 occurs in tumors in response to IFN-*γ*, which is secreted by activated CD8^+^ T cells, while the expression of IDO1 is increased, CD8^+^ T cell response will be suppressed, resulting in tumor immune evasion and tumor growth. However, miR-448 downregulates IDO1 protein expression, and the feedback of IDO1 to CD8^+^ T cells will be impaired. Thus, the number of CD8^+^ T cells in the tumor microenvironment will be increased, resulting in tumor rejection

Upregulation of IDO1 occurs in CRC in response to IFN-*γ,* which is secreted by CD8^+^ T cells [44], while IDO1 suppresses the CD8^+^ T cell response, suggesting a possible negative feedback loop to regulate T cell activation, as reported in APCs [45]. One hypothesis is that, in colon cancer, IDO1 expression appears to be higher due to increased CD8^+^ T cell infiltration. In this case, higher expression of IDO1 may be a surrogate for a stronger spontaneous anti-tumor immune response to exert protective effects [14], resulting in a better prognosis. The other hypothesis is that, early diagnosis combined with effective treatment always result in improved survival in patients with colon cancer, thus the treatment type is an important factor affecting the overall survival of patients with early-stage colon cancer. In fact, IDO1 itself is not helpful all the time, even in these immune-responsive patients. The patients might have a favorable prognosis if IDO1 was blocked. High IDO1 levels suppress the CD8^+^ T cell response in these patients, resulting in tumor immune escape (Fig. 7). Furthermore, we found that the protein and mRNA expression patterns of IDO1 are different in colon cancer. We hypothesize that miRNAs may downregulate IDO1 expression at the posttranscriptional level. miRNAs may play an important role in the immune equilibrium of IDO1 and CD8^+^ T cells in the colon cancer microenvironment.

Some miRNAs can regulate several genes to influence tumorigenesis. Recent studies have shown that some miRNAs are dysregulated in colon cancer [16–19]. The expression of miR-448 in colon cancer tissues is significantly downregulated compared with that in adjacent normal tissues [46, 47]. miR-448 exerts tumor suppressor roles in colon cancer [46, 47]. Here, we validated that miR-448 targeted IDO1 in colon cancer. Notably, we proved that miR-448 suppressed the apoptosis of CD8^+^ T cells by targeting IDO1 in human colon cancer. IDO1 will be significantly induced by CD8^+^ T cells [14], but miR-448 can remarkably downregulate IDO1 protein expression. Therefore, the feedback of IDO1 to the CD8^+^ T cell response will be impaired (Fig. 7), and the number of CD8^+^ T cells in the tumor microenvironment will not decrease. As a result, CD8^+^ T cells exert cytotoxic T lymphocyte effector functions, including the release of cytokines to mediate the deposition of cytotoxic granules in the vicinity of target-cell membranes to induce tumor cell apoptosis [48, 49]. ISH results showed that miR-448 suppressed the protein expression of IDO1 in human colon cancer (Fig. 5g). Of note, miR-448 substantially reduced IFN-*γ*-induced IDO1 expression in HT-29 and HCT-116 cells, but it was difficult to detect miR-448 inhibition to IDO1 without IFN-*γ* stimulation by WB (Additional file 1: Figure S5d). So the suppressive effect of miR-448 on IDO1 expression will be aggravated in IFN-*γ*^high^ tumor microenvironment, which means that in “immune hot” (highly infiltrated T cells) tumors [50], IDO1 level will be elevated faster in these miR-448^low^ colon cancer patients compared with miR-448^high^ colon cancer patients, and IDO1 will substantially suppress CD8^+^ T cells during tumor progression.

We believe that miR-448 is not only an important regulator of IDO1 related negative feedback, but also a potential biomarker for IDO1-related immunotherapy. Compared with miR-448^high^ colon cancer patients, IDO1 level will be elevated faster in these miR-448^low^ colon cancer patients when they receive T cell related therapy (CAR-T, anti-PD-1) due to the lack of endogenous miR-448 inhibition to IDO1. Thus, it is more necessary to suppress IDO1, when these miR-448^low^ colon cancer patients receive T cell related therapy. Therefore, we suggest that miR-448 targets IDO1 in the tumor microenvironment and may be a potential biomarker for IDO1-related colon cancer immunotherapy.

## Conclusions

In summary, stable IDO1 overexpression has no effect on subcutaneous tumor growth in immune-deficient nude mice but promotes tumor growth in immune competent mice by suppressing the CD8^+^ T cell response. IDO1 was a credible target of miR-448 which, as a tumor-suppressive miRNA, enhances the CD8^+^ T cell response by inhibiting IDO1 expression. Therefore, miR-448 targets IDO1 in the tumor microenvironment and may be a potential biomarker for colon cancer. Our results provide a theoretical basis for the development of new immunotherapy for the treatment of colon cancer.

## Additional file

Additional file 1: **Figure S1.** Mice body weight and immunohistochemical staining in sections of mouse tissues. **Figure S2.** Gating strategies of FACS, and the phenotypic and functional features of the T cell subsets. **Figure S3.** Representative IHC staining intensity of IDO1 in human colon cancer tissues and adjacent noncancerous tissues. **Figure S4.** miR-30a-5p downregulates IDO1 expression. **Figure S5.** IDO1 is induced by IFN-*γ* and suppressed by miR-30a-5p. **Figure S6.** Representative ISH staining intensity of miR-448 in human colon cancer tissues and adjacent noncancerous tissues. **Figure S7.** IFN-*γ* promotes IDO1 enzyme function and miR-448 suppresses IDO1 enzyme function. **Figure S8.** The schema of the major steps in our study. **Figure S9.** IDO1 expression in relation to the survival of patients with colon cancer. **Table S1.** Quantitative reverse transcription polymerase chain reaction primers. **Table S2.** Antibodies for flow cytometry analysis in mouse tumor tissues. **Table S3.** Correlations of IDO1 mRNA levels with clinicopathological variables in colon cancer. **Table S4.** Correlations of IDO1 protein levels with clinicopathological variables in colon cancer. **Table S5.** The concentration of IDO1 in the culture medium from HCT-116 cells and HT-29 cells (transfection with miR-448 mimic or negative control followed by IFN-*γ* for 24 h). (DOCX 6830 kb)

## Data Availability

All data generated or analyzed during this study are included in this article and its Additional file 1.

## Abbreviations

ANOVA: One-way analysis of variance
APC: Antigen-presenting cells
ATCC: American Type Culture Collection
CRC: Colorectal cancer
FACS: Fluorescence-activated cell sorting
FITC: Fluorescein isothiocyanate
Foxp3: Forkhead box P3
GAPDH: Glyceraldehyde-3-phosphate dehydrogenase
HPLC: High performance liquid chromatography
IDO1: Indoleamine 2,3 dioxygenase 1
IFN: Inteferon
IHC: Immunohistochemistry
ISH: In situ hybridization
Kyn: Kynurenine
miR-30a-5p: MicroRNA 30a-5p
miR-448: MicroRNA 448
MSI-H: Microsatellite instability-high
NK: Natural killer
qRT-PCR: Quantitative real-time polymerase chain reaction
RNA: Ribonucleic acid
RTCA: Real-time cell analysis
TCGA: The Cancer Genome Atlas
TMA: Tissue microarray
Trp: Tryptophan
WB: Western blotting
WT: Wild-type
