# Supplementary material for: miR-448 targets IDO1 and regulates CD8+ T cell response in human colon cancer
Source: J Immunother Cancer. 2019 Aug 7;7:210. doi: 10.1186/s40425-019-0691-0 (PMC6686234; doi:10.1186/s40425-019-0691-0)
Supplement: Supplementary file 1 — Figure S1. Mice body weight and immunohistochemical staining in sections of mouse tissues. Figure S2. Gating strategies of FACS, and the phenotypic and functional features of the T cell subsets. Figure S3. Representative IHC staining intensity of IDO1 in human colon cancer tissues and adjacent noncancerous tissues. Figure S4. miR-30a-5p downregulates IDO1 expression. Figure S5. IDO1 is induced by IFN-γ and suppressed by miR-30a-5p. Figure S6. Representative ISH staining intensity of miR-448 in human colon cancer tissues and adjacent noncancerous tissues. Figure S7. IFN-γ promotes IDO1 enzyme function and miR-448 suppresses IDO1 enzyme function. Figure S8. The schema of the major steps in our study. Figure S9. IDO1 expression in relation to the survival of patients with colon cancer. Table S1. Quantitative reverse transcription polymerase chain reaction primers. Table S2. Antibodies for flow cytometry analysis in mouse tumor tissues. Table S3. Correlations of IDO1 mRNA levels with clinicopathological variables in colon cancer. Table S4. Correlations of IDO1 protein levels with clinicopathological variables in colon cancer. Table S5. The concentration of IDO1 in the culture medium from HCT-116 cells and HT-29 cells (transfection with miR-448 mimic or negative control followed by IFN-γ for 24 h). (DOCX 6830 kb) [file 40425_2019_691_MOESM1_ESM.docx]

**miR-448 targets IDO1 and regulates CD8^+^ T cell response in human colon cancer**

Qiong Lou^1,2^, Ruixian Liu^1^, Xiangling Yang^1^, Weiqian Li^1^, Lanlan Huang^1^, Lili Wei^2^, Huiliu Tan^2^, Nanlin Xiang^1^, Kawo Chan^1^, Junxiong Chen^1,2^, Huanliang Liu^1,2*^

^1^Guangdong Provincial Key Laboratory of Colorectal and Pelvic Floor Diseases, Guangdong Institute of Gastroenterology, The Sixth Affiliated Hospital, Sun Yat-sen University, Guangzhou, Guangdong 510655, China

^2^Department of Clinical Laboratory, The Sixth Affiliated Hospital, Sun Yat-sen University, Guangzhou, Guangdong 510655, China

*Correspondence: liuhuanl@mail.sysu.edu.cn

**Figures**

**
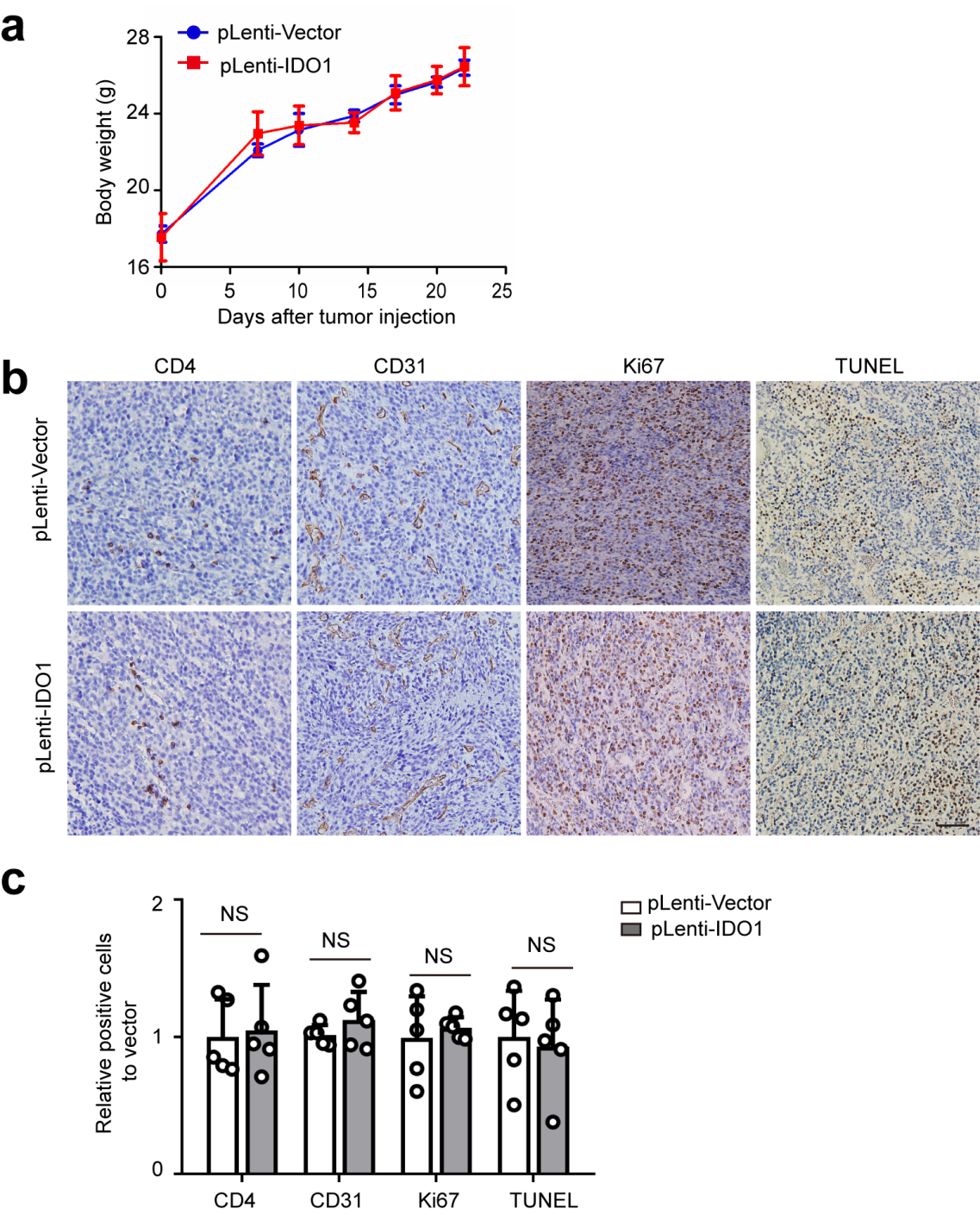
**

**Figure S1.** **Mice body weight and immunohistochemical staining in sections of mice tissues. (a)** Average body weight of BALB/c mice after inoculation of CT26 cells with stable IDO1 overexpression (pLenti-IDO1) or with vector control (pLenti-Vector). **(b)** Representative pictures and **(c)** quantitative data of immunohistochemical staining for CD4, CD31, Ki67 and TUNEL staining in sections of mice tumor tissues from the pLenti-IDO1 group and the pLenti-Vector group. Scale bars: 50 µm. **(a, c)** Mean ± SEM. **(a)** n = 6; **(b, c)** n = 5**.** **(a)** One-way ANOVA and **(c)** two-tailed Student’s t-test were performed for statistical analysis; NS: not significant.


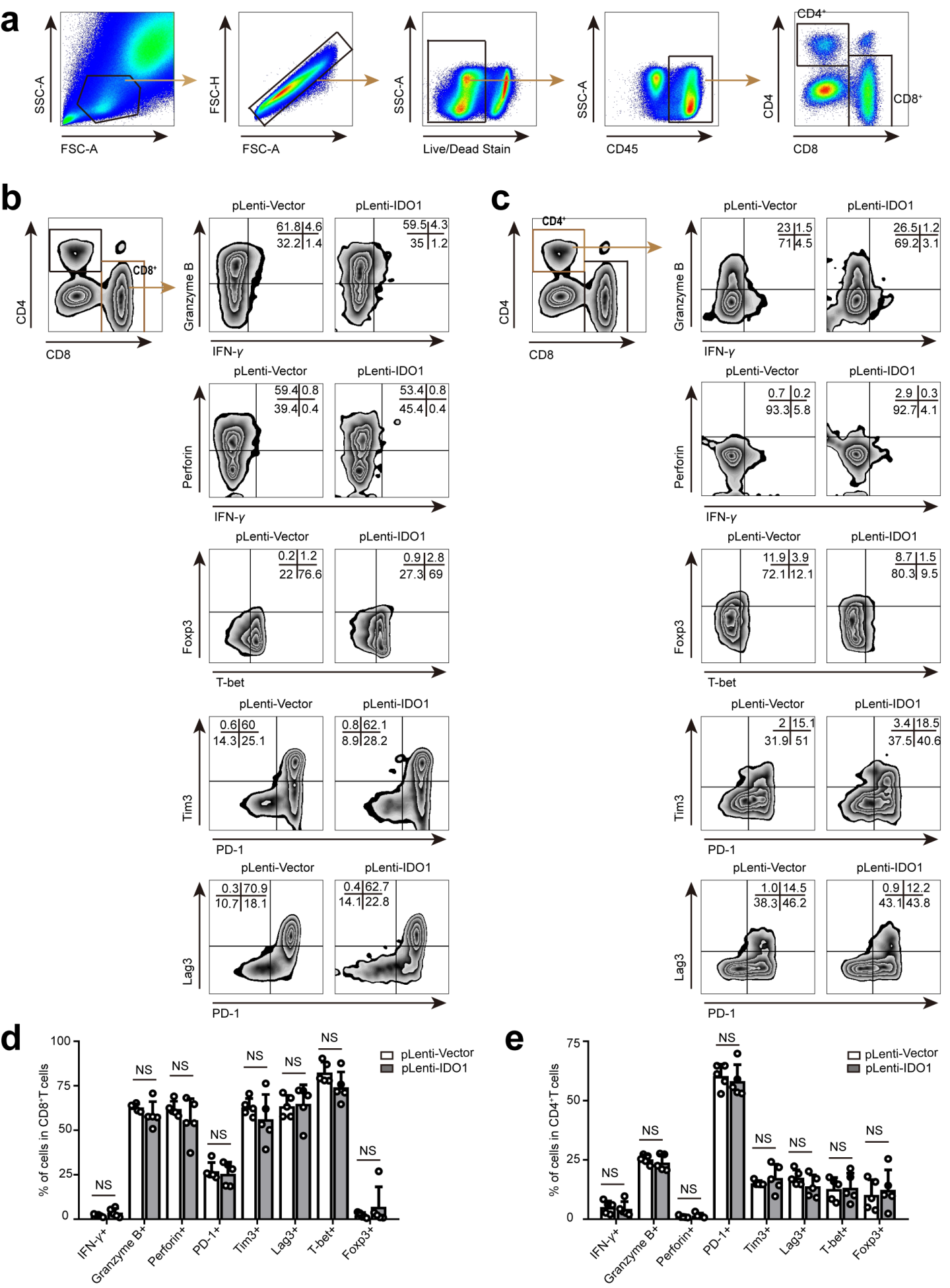


**Figure S2. Gating strategies of FACS, and the phenotypic and functional features of the T cell subsets.** FACS analysis of mice tumor tissues were from the pLenti-IDO1 group or the pLenti-Vector group. **(a)** Cells isolated from tumor tissues were first gated for lymphocytes (SSC-A vs. FSC-A) and then for singlets (FSC-H vs. FSC-A). The singlets gate was further analyzed for their uptake of the Zombie Yellow^TM^ Live/Dead stain. The samples were then analyzed by gating on the live population and CD45^+^ cells were selected for further characterization of CD8^+^ and CD4^+^ T cell subsets. The expression pattern analysis of IFN-*γ*, Granzyme B, perforin, Foxp3, T-bet, PD-1, Tim3 and Lag3 in **(b)** CD8^+^ T cells or **(c)** CD4^+^ T cells was given as representative of the final gating. IFN-*γ*, Granzyme B, perforin, Foxp3, T-bet, PD-1, Tim3 and Lag3 producing **(d)** CD8^+^ T or **(e)** CD4^+^ T cells were quantified. **(d, e)** Mean ± SD. **(b-e)** n = 5. **(d, e)** Two-tailed Student’s t-test was performed for statistical analysis; NS: not significant.


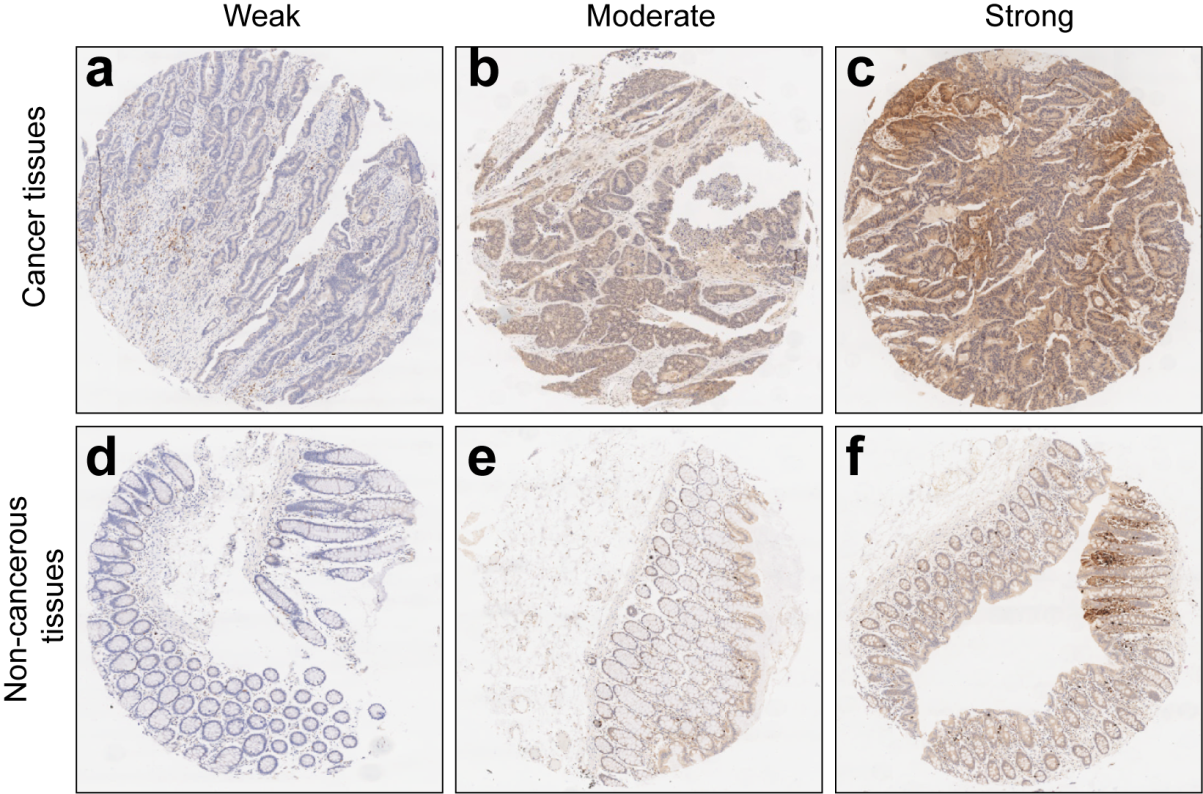


**Figure S3.** **Representative IHC staining intensity of IDO1 in human colon cancer tissues and adjacent noncancerous tissues.** The staining intensity of IDO1 was divided into tertiles according to the score using Image-Pro Plus 6.0 software. The staining intensity in the first tertile, second tertile, and third tertile was as weak expression, moderate expression and strong expression, respectively. Representative pictures of **(a, d)** weak expression, **(b, e)** moderate expression, and **(c, f)** strong expression.


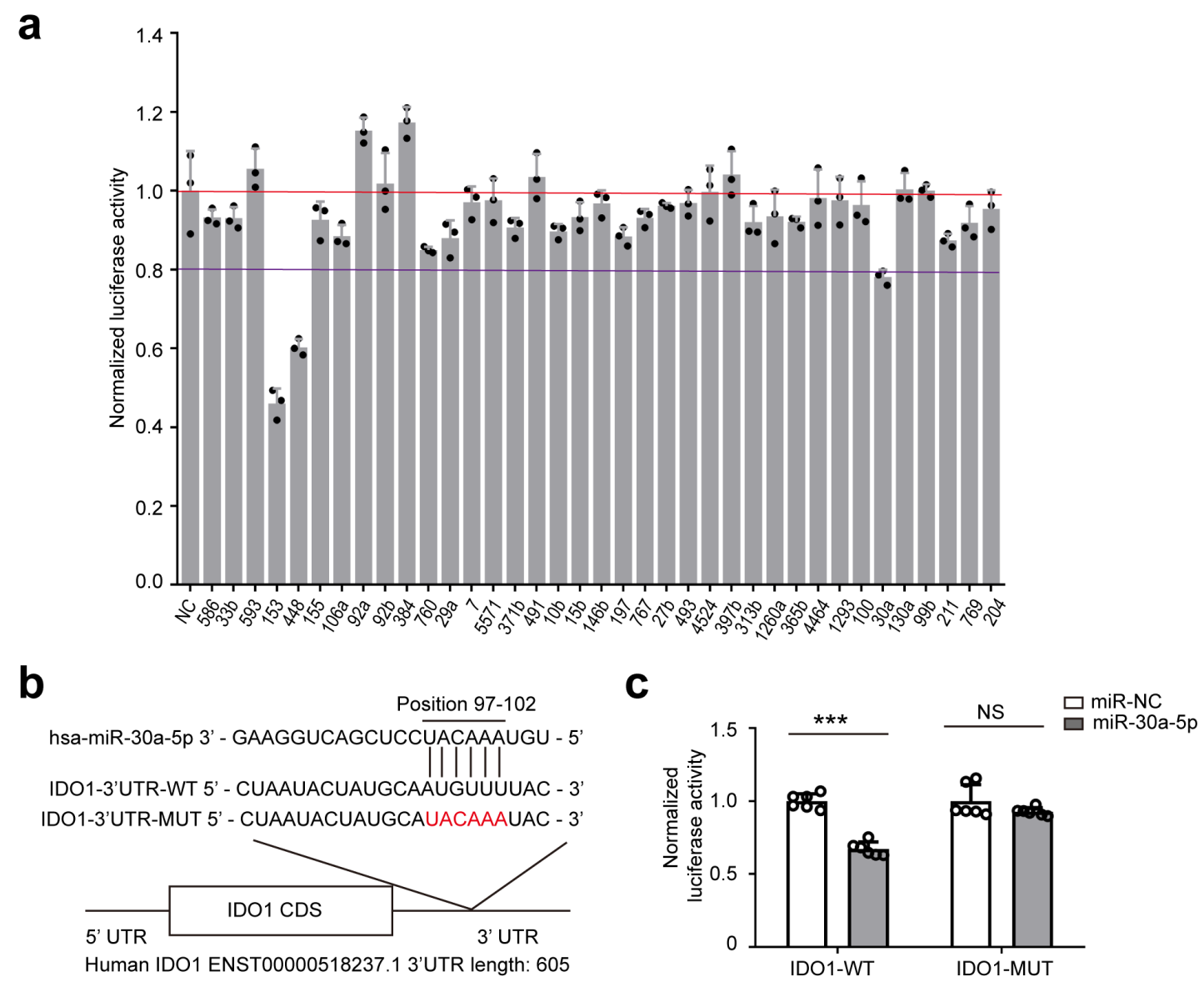


**Figure S4.** **miR-30a-5p downregulates IDO1 expression. (a)** Luciferase reporter containing wild-type (WT) IDO1 3’ UTR was cotransfected with 40 miRNA mimics or a negative control (NC) into HT-29 cells. Relative firefly luciferase expression was normalized to Renilla luciferase. NC: miRNA mimic negative control, 586: microRNA-586 mimic, etc. (**b)** The binding site of miR-30a-5p in IDO1 mRNA. **(c)** The relative luciferase activity in HCT-116 cells cotransfected with miR-30a-5p mimics and IDO1-WT, or cotransfected with miR-30a-5p mimics and IDO1-MUT. **(a)** Mean ± SD; **(c)** mean ± SEM. **(a)** n = 3; **(c)** n = 6 measurements from two biological replicates performed in triplicate. **(a, c)** Two-tailed Student’s t-test was performed for statistical analysis; ***P < 0.001, NS: not significant. miR-NC: miRNA mimic negative control; miR-30a-5p: miRNA-30a-5p mimic.

**
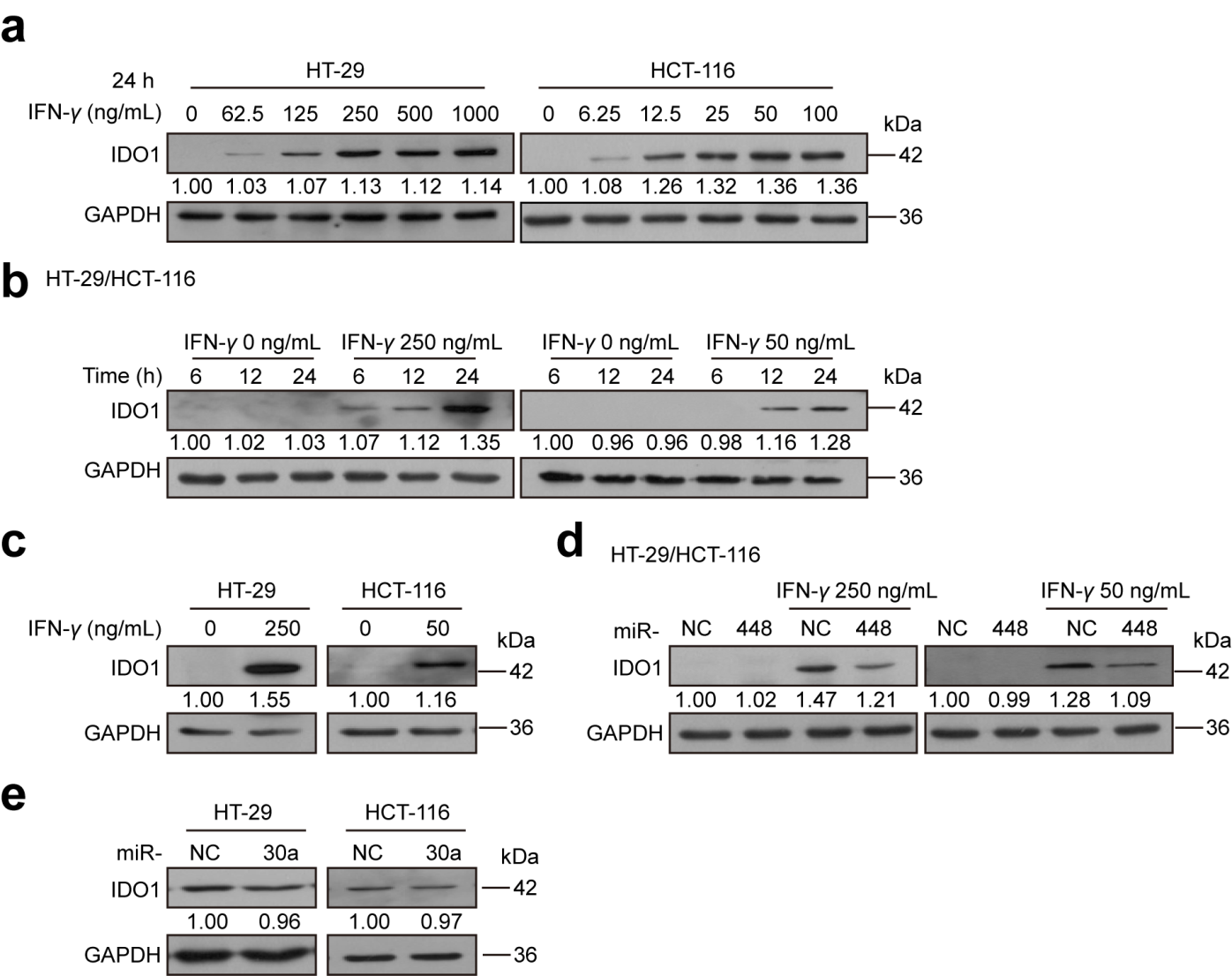
**

**Figure S5.** **IDO1 is induced by IFN-*γ* and suppressed by miR-30a-5p. (a)** IDO1 protein expression in HT-29 and HCT-116 cells stimulated by 0-1000 ng/mL or 0-100 ng/mL IFN-*γ* for 24 h, respectivetly. **(b)** IDO1 protein expression in HT-29 and HCT-116 cells treated with 250 ng/mL and 50 ng/mL IFN-*γ*, respectively, for 6-24 h. **(c)** The protein expression of IDO1 in HT-29 and HCT-116 cells treated with 250 ng/mL or 50 ng/mL IFN-*γ*, respectively, for 24 h. **(d)** IDO1 protein expression in HT-29 and HCT-116 cells transfected with miR-448 mimics or negative control without IFN-*γ* treatment, and transfected with miR-448 or negative control with IFN-*γ* treatment for 24 h. **(e)** IDO1 protein expression in HT-29 and HCT-116 cells transfected with miR-30a-5p mimics or negative control followed by IFN-*γ* treatment for 24 h. GAPDH was served as the internal control. **(a-e)** Images are representative of three biological replicates.

**
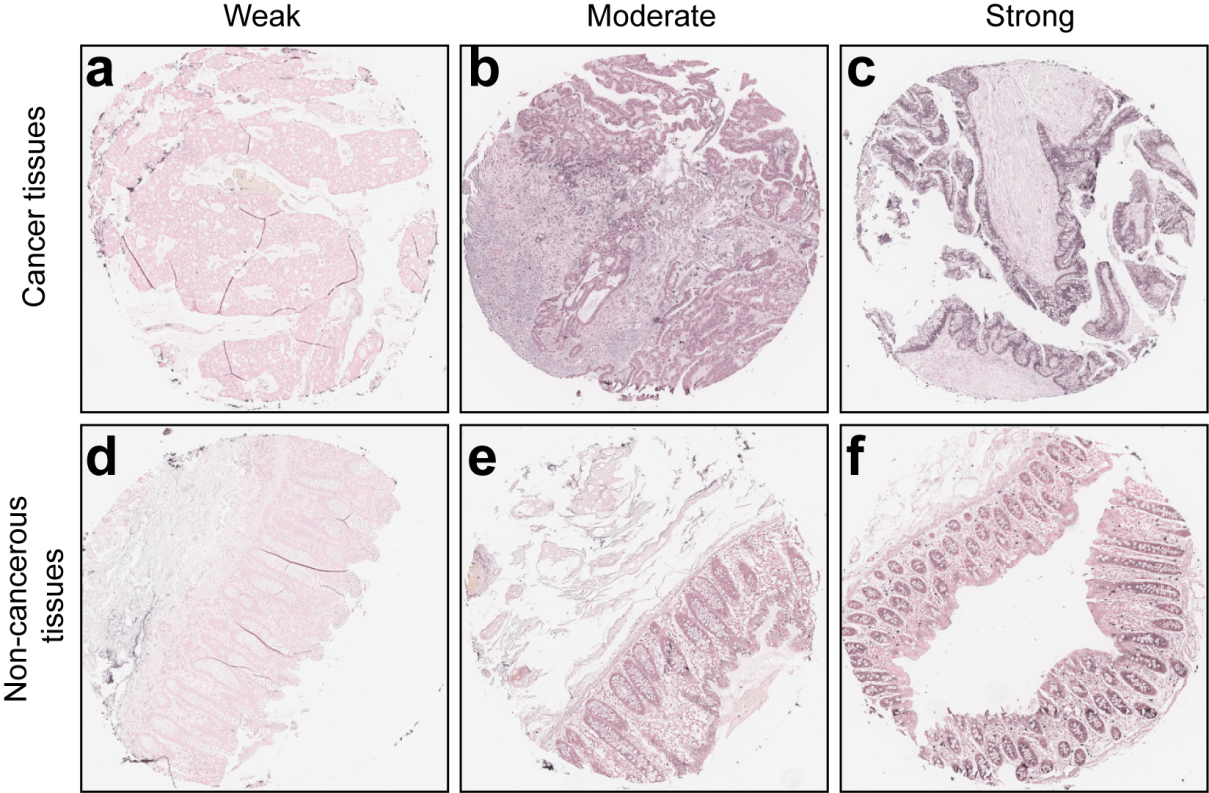
**

**Figure S6.** **Representative** **ISH staining intensity of miR-448 in human colon cancer tissues and adjacent noncancerous tissues.** The staining intensity of miR-448 was divided into tertiles according to the score using Image-Pro Plus 6.0 software. The staining intensity in the first tertile, second tertile, and third tertile was as weak expression, moderate expression and strong expression, respectively. Representative pictures of **(a, d)** weak expression, **(b, e)** moderate expression and **(c, f)** strong expression.

**
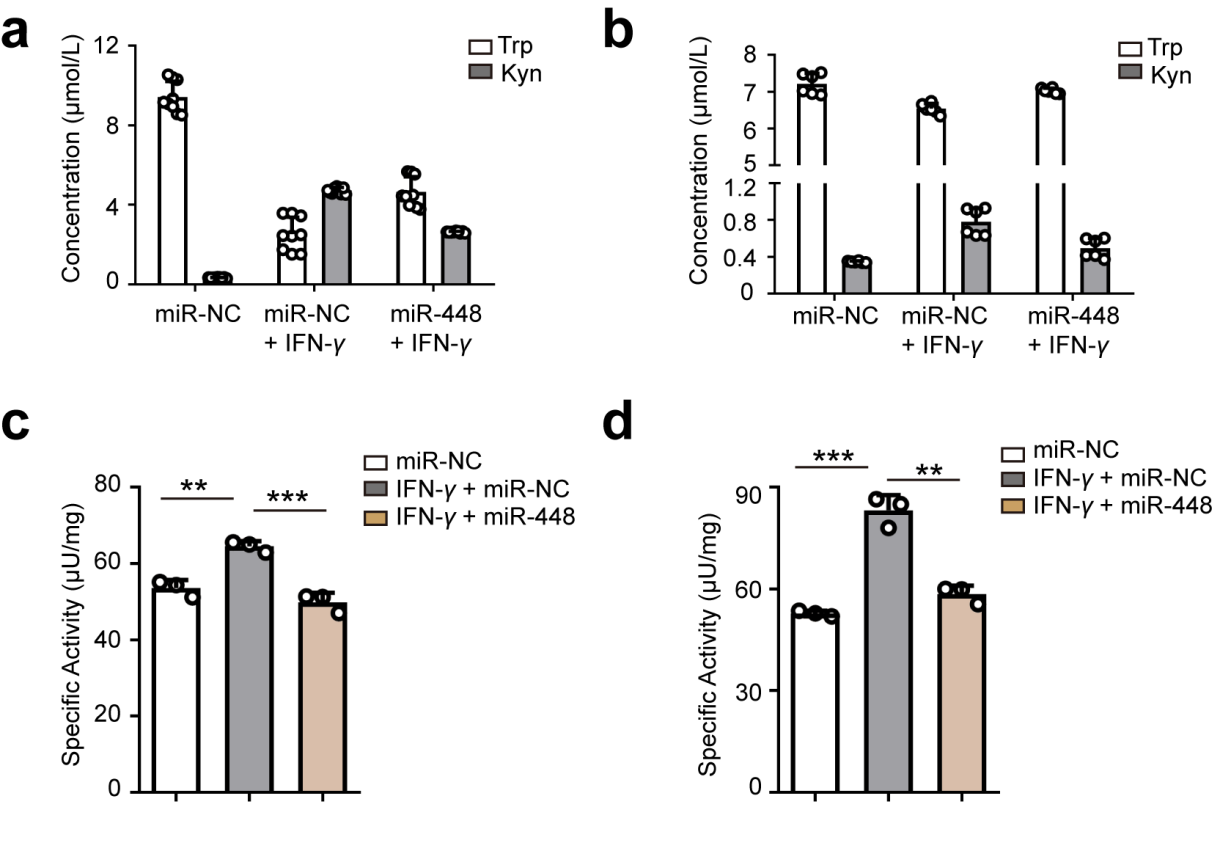
**

**Figure S7. IFN-*γ* promotes IDO1 enzyme function and** **miR-448 suppresses IDO1 enzyme function.** Concentration of Trp and Kyn in the culture medium from **(a)** HT-29 and **(b)** HCT-116 cells transfected with miR-NC, or transfected with miR-NC followed by IFN-*γ* treatment, or transfected with miR-448 followed by IFN-*γ* treatment were determined by HPLC. IDO1 function in cells lysates (30 μL) from **(c)** HT-29 and **(d)** HCT-116 cells transfected with miR-NC, or transfected with miR-NC followed by IFN-*γ* treatment, or transfected with miR-448 followed by IFN-*γ* treatment for 24 h were determined using IDO1 activity assay kit (50 ng/mL IFN-*γ* for HCT-116 and 250 ng/mL IFN-*γ* for HT-29). **(a-d)** Mean ± SEM. **(a)** n = 9 measurements from three biological replicates performed in triplicate**;** **(b)** n = 6 measurements from two biological replicates performed in triplicate**;** **(c, d)** n = 3 biologically independent experiments**. (a-d)** Two-tailed Student’s t-test was performed for statistical analysis; **P<0.01,***P<0.001. miR-NC: miRNA mimic negative control; miR-448: miRNA-448 mimic.


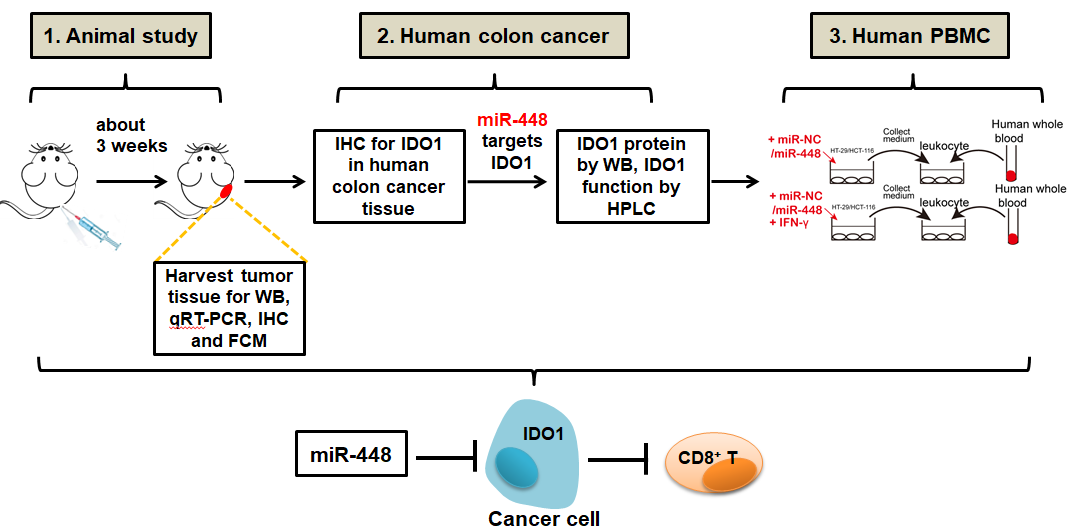


**Figure S8. The schema of the major steps in our study.** The major steps includes animal study, the study of human colon cancer and the study of human PBMC. (1) Animal study: male BALB/c mice were subcutaneously inoculated in the right flank with 4 × 10^5^ CT26 cells with IDO1 stable overexpression or not. About 3 weeks posttransplantation, CT26 tumors were harvested for WB, qRT-PCR, IHC and FACS. (2) The study of human colon cancer: IHC of IDO1 was performed in human colon cancer tissues and adjacent noncancerous tissues; dual luciferase reporter containing wild-type (WT) IDO1 3’ UTR was cotransfected with 40 miRNA mimics or a negative control (NC) into HCT-116 or HT-29 cells to screen miRNAs targeting IDO1; IDO1 protein expression was detected by WB, and IDO1 function was detected by HPLC. (3) The study of human PBMC: human leukocytes were isolated from peripheral blood collected from volunteers. miR-448 mimic or NC was transfected into HT-29 and HCT-116 cells in the presence or absence of 250 ng/mL or 50 ng/mL IFN-*γ*, respectively. We collected the cell culture supernatant after treatment with IFN-*γ* for 24 h as the conditional medium (CM). One hundred microliters of leukocytes were seeded into a 96-well plate, after which 100 μL of different CM was added into the 96-well plate and cultured for 48 h. Then CD8^+^ T apoptosis in leukocytes was detected by flow cytometer.

**
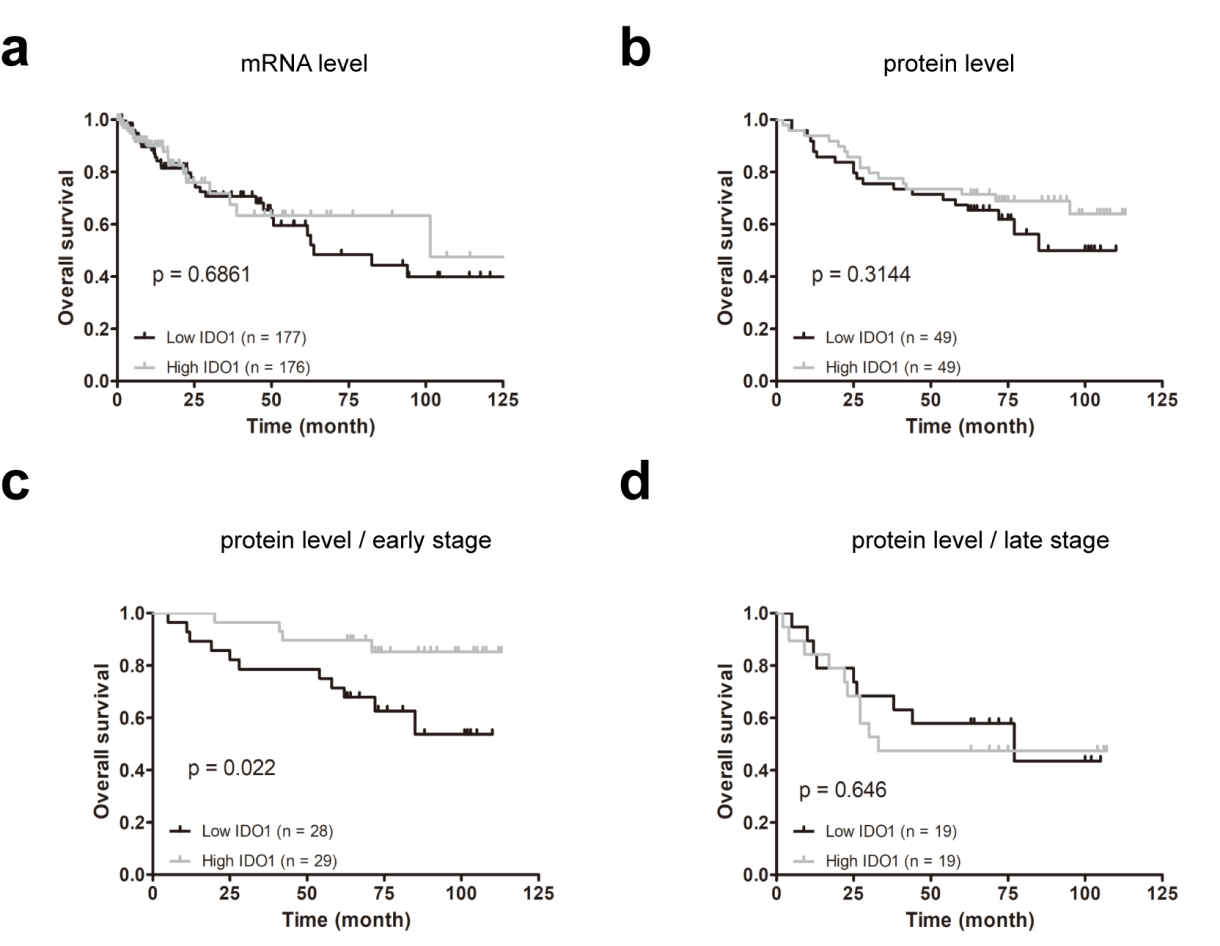
**

**Figure S9. IDO1 expression in relation to the survival of patients with colon cancer.** **(a)** Kaplan–Meier analysis of overall survival according to low and high IDO1 mRNA expression in 353 colon cancer patients downloaded from the TCGA database. **(b)** Kaplan–Meier analysis of overall survival according to low and high IDO1 protein expression in 100 colon cancer patients as assessed by IHC. **(c)** Kaplan–Meier analysis of overall survival according to low and high IDO1 protein expression in 57 colon cancer patients with early-stage disease (stage I + stage II). **(d)** Kaplan–Meier analysis of overall survival according to low and high IDO1 protein expression in 38 colon cancer patients with late-stage disease (stage III + stage IV). **(a-b)** The log-rank test was performed for statistical analysis.

**Table**

**Table S1**. Quantitative reverse transcription polymerase chain reaction primers.

| **Gene** | **Forward primer** | **Reverse primer** |
| --- | --- | --- |
| Mouse IDO1 | AAGGGCTTCTTCCTCGTCTC | AAAAACGTGTCTGGGTCCAC |
| Mouse GAPDH | GACATCAAGAAGGTGGTGAAGGAG | ATACCAGGAAATGAGCTTGACAAA |
|  | | |

**Table S2**. Antibodies for flow cytometry analysis in mouse tumor tissues.

| **Name** | **Company** | **Product code** |
| --- | --- | --- |
| Pacific Blue™ anti-mouse CD45 | BioLegend | Cat# 103126 |
| PerCP/Cyanine 5.5 anti-mouse CD4 | BioLegend | Cat# 100540 |
| Brilliant Violet 421™ anti-mouse FOXP3 | BioLegend | Cat# 126419 |
| Brilliant Violet 605™ anti-T-bet | BioLegend | Cat# 644817 |
| APC anti-mouse Perforin | BioLegend | Cat# 154404 |
| FITC anti-human/mouse Granzyme B Recombinant | BioLegend | Cat# 372206 |
| PE/Dazzle™ 594 anti-mouse CD279 (PD-1) | BioLegend | Cat# 109115 |
| Brilliant Violet 711™ anti-mouse CD366 (Tim-3) | BioLegend | Cat# 119727 |
| Brilliant Violet 785™ anti-mouse CD223 (LAG-3) | BioLegend | Cat# 125219 |
| Zombie Yellow^TM^ fixable viability kit | BioLegend | Cat# 423103 |
| Ms CD8A Horizon V500 53-6.7 | BD Bioscience | Cat# 560778 |
| BV650 Rat anti-mouse IFN-*γ* | BD Bioscience | Cat# 563854 |
| Brilliant Violet 421™ Rat IgG2b, κ Isotype Ctrl | BioLegend | Cat# 400655 |
| Brilliant Violet 605™ Mouse IgG1, κ Isotype Ctrl | BioLegend | Cat# 400161 |
| FITC Mouse IgG1, κ Isotype Ctrl (ICFC) | BioLegend | Cat# 400137 |
| PE/Dazzle™ 594 Rat IgG2b, κ Isotype Ctrl | BioLegend | Cat# 400659 |
| Brilliant Violet 785™ Rat IgG1, κ Isotype Ctrl | BioLegend | Cat# 400443 |
| Brilliant Violet 711™ Rat IgG2a, κ Isotype Ctrl | BioLegend | Cat# 400551 |
| APC Rat IgG2a, κ Isotype Ctrl | BioLegend | Cat# 400511 |
| Rat lgG1 Kpa itCI BV650 R3-34 | BD Bioscience | Cat# 563848 |

**Table S3.** Correlations of IDO1 mRNA levels with clinicopathological variables in colon cancer.

| Factors | Number of cases | IDO1 mRNA expression | | P value |
| --- | --- | --- | --- | --- |
|  |  | Low | High |  |
| Age |  |  |  | 0.144 |
| <60 | 126 | 70 (55.6%) | 56 (44.4%) |  |
| ≥60 | 331 | 158 (47.7%) | 173 (52.3%) |  |
| Gender |  |  |  | 0.64 |
| Female | 216 | 105 (48.6%) | 111(51.4%) |  |
| Male | 241 | 123 (51.0%) | 118 (48.9%) |  |
| pT |  |  |  | 0.543 |
| T1+T2 | 88 | 42 (47.7%) | 46 (52.3%) |  |
| T3+T4 | 368 | 186 (50.5%) | 182 (49.5%) |  |
| pN |  |  |  | 0.129 |
| N0 | 269 | 126 (46.8%) | 143 (53.2%) |  |
| N1+N2 | 188 | 102 (54.3%) | 86 (45.7%) |  |
| pM |  |  |  | 0.005^*^ |
| M0 | 335 | 154 (46.0%) | 181(54.0%) |  |
| M1 | 115 | 72 (62.6%) | 43 (37.4%) |  |
| TNM stage |  |  |  | 0.068 |
| I+II | 253 | 115 (45.5%) | 138 (54.5%) |  |
| III+IV | 193 | 108 (56.0%) | 85 (44.0%) |  |

pT represents depth of tumor invasion; pN represents lymph node metastases; pM represents

distant metastasis. ^*^ P < 0.05.

**Table S4**. Correlations of IDO1 protein levels with clinicopathological variables in colon cancer.

| Factors | Number of cases | IDO1 protein expression | | P value |
| --- | --- | --- | --- | --- |
|  |  | Low | High |  |
| Age |  |  |  | 0.53 |
| <60 | 49 | 20 (40.8%) | 29 (59.2%) |  |
| ≥60 | 49 | 16 (32.7%) | 33 (67.3%) |  |
| Gender |  |  |  | 0.307 |
| Female | 49 | 18 (36.7%) | 31 (63.3%) |  |
| Male | 49 | 24 (49.0%) | 25 (51.0%) |  |
| pT |  |  |  | 0.242 |
| T1+T2 | 47 | 3 (6.4%) | 44 (93.6%) |  |
| T3+T4 | 46 | 0 (0%) | 46 (100%) |  |
| pN |  |  |  | 0.293 |
| N0 | 49 | 10 (20.4%) | 10 (20.4%) |  |
| N1+N2 | 49 | 16 (32.7%) | 6 (12.2%) |  |
| pM |  |  |  | 1 |
| M0 | 49 | 48 (98.0%) | 1 (2.0%) |  |
| M1 | 49 | 49 (100.0%) | 0 (0.0%) |  |
| TNM stage |  |  |  | 0.964 |
| I+II | 48 | 29 (60.4%) | 19 (39.6%) |  |
| III+IV | 47 | 28 (60.9%) | 18 (39.1%) |  |

pT represents depth of tumor invasion; pN represents lymph node metastases; pM represents

distant metastasis.

**Table S5**. The concentration of IDO1 in the culture medium from HCT-116 cells and HT-29 cells (transfection with miR-448 mimic or negative control followed by IFN-*γ* for 24 h).

|  | **Group** | **Concentration (IU/mL)** | | | **MEAN** | **SD** |
| --- | --- | --- | --- | --- | --- | --- |
| HCT-116 | miR-NC | 0.0397 | 0.0381 | 0.0381 | 0.0387 | 0.0009 |
|  | miR-NC + IFN-*γ* | 0.0410 | 0.0409 | 0.0355 | 0.0391 | 0.0032 |
|  | miR-448 + IFN-*γ* | 0.0395 | 0.0368 | 0.0371 | 0.0378 | 0.0015 |
| HT-29 | miR-NC | 0.0379 | 0.0367 | 0.0362 | 0.0369 | 0.0009 |
|  | miR-NC + IFN-*γ* | 0.0357 | 0.0367 | 0.0433 | 0.0386 | 0.0041 |
|  | miR-448 + IFN-*γ* | 0.0386 | 0.0379 | 0.0379 | 0.0381 | 0.0004 |
